# In Depth Characterization of the Promoter Proximal Proteome of Single Copy Locus *FOXP2*

**DOI:** 10.1016/j.mcpro.2026.101570

**Published:** 2026-04-21

**Authors:** Tim M.G. MacKenzie, Lucia Ramirez, Ruiqi Jian, Lihua Jiang, Michael P. Snyder

**Affiliations:** Department of Genetics, Stanford University, Stanford, California, USA

**Keywords:** transcription factors, proximity labeling, locus-specific chromatin proteomics, tribrid mass spectrometer, subcellular spatial proteomics, TMT-RTS-SPS-MS3, FOXP2

## Abstract

Identifying the proteins that interact with sequence-defined chromatin segments is a critical step in understanding gene expression. Most procedures perform bulk analysis of samples to identify the general interactions that occur in a cellular population and thereby do not detect the proteins that operate at a single locus. To circumvent this limitation, we developed a modified method that uses genetically targeted proximity labeling with dCas9-APEX2 to specifically biotinylate the promoter proximal proteome of the single copy locus *FOXP2* in live HEK293 cells. After capture of the tagged proteins with streptavidin and isobaric labeling of the peptides produced from on-bead digestion with tandem mass tags, we used quantitative 2D-LC-MS3 on a tribrid mass spectrometer to identify 373 significantly enriched proteins at the active promoter relative to control samples (Storey-*q* < 0.05, fold change > 1.2). These proteins were enriched for transcription factors (TFs) and components of the spliceosome. To validate our candidate transcriptional regulators, we utilized computationally predicted TF binding and the >200 ChIP-Seq experiments performed in HEK293 cells by Encyclopedia of DNA Elements. In addition to validating dozens of candidate TFs as binders of the targeted genomic locus, we newly identify IRF2BP2 and glucocorticoid signaling as negative regulators of *FOXP2* transcription, suggesting they each play a key role in *FOXP2* gene expression. We further demonstrate that MS detects approximately one third of both binders and nonbinders, with more highly expressed genes significantly more likely to be detected regardless of binding status or locus specificity. Encyclopedia of DNA Elements ChIP-Seq binders not detected by MS show significantly lower expression compared to nonbinders only at the targeted *FOXP2* promoter and not at off target loci.

## Highlights

• dCas9-APEX2 proximity labeling reveals promoter proteome of single copy locus *FOXP2.*
• Deep proteome coverage with 2D-LC and TMT-RTS-SPS-MS3 on a tribrid instrument.
• Multi-omic integration including ENCODE ChIP-Seq to confirm dozens of locus binders.
• IRF2BP2 and glucocorticoid signaling discovered as negative regulators of *FOXP2.*
• Lowly expressed transcription factors significantly less likely to be detected by MS.

## Graphical Abstract

**Figure:**
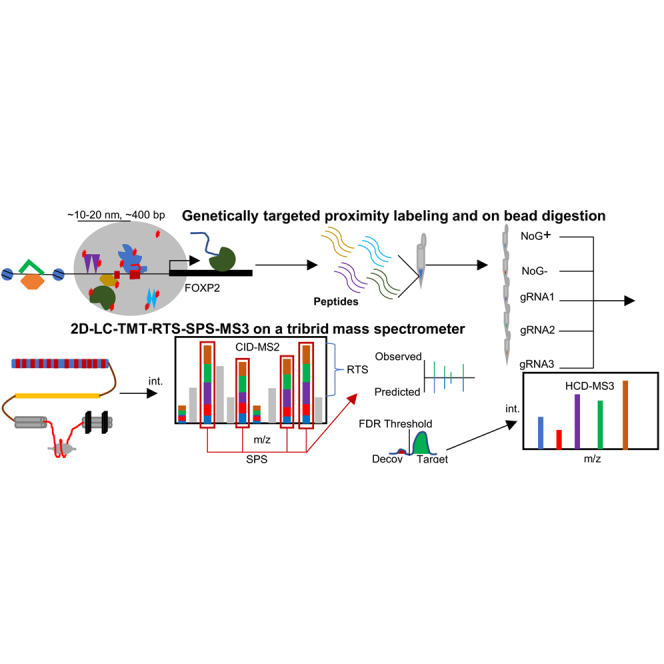

## In Brief

Genetically targeted proximity labeling with dCas9-APEX2 coupled to quantitative mass spectrometry proteomics *via* 2D-LC-TMT-RTS-SPS-MS3 on a tribrid mass spectrometer uncovers promoter interactors at the single copy locus *FOXP2*. Candidate proteins are cross-validated with extensive multi-omic integration, including computationally predicted locus binders and hundreds of ChIP-Seq experiments performed by ENCODE. IRF2BP2 and glucocorticoid signaling are revealed as regulators of *FOXP2* expression. MS transcription factor detection is significantly correlated with expression levels, with more lowly expressed proteins less likely to be detected.

Deciphering the factors that regulate gene expression at important loci is crucial for linking genotype and phenotype. These transcription factors (TFs) bind to DNA and recruit transcriptional machinery and chromatin remodeling proteins to impact gene expression. The composition of proteins at both promoters and enhancers integrates environmental and cell-intrinsic signals to determine the activation or silencing of a gene at steady state and in response to external cues (1, 2, 3). Determining the proteins proximal to a gene promoter is a key step in building a mechanistic understanding of gene expression and developing experimental interventions in the process.

There is a growing appreciation for the role of alternative promoter selection in regulating gene expression (4, 5, 6, 7, 8, 9). Results from both long read (10) and traditional short read (11) sequencing have indicated that alternative promoters resulting in different transcription start sites (TSSs) and 5′ UTRs are the major driver of transcriptome diversity in many cancers and can provide a better patient prognosis than gene-level expression. The choice of TSS and the resulting 5′ UTR can impact the translation efficiency of the resulting gene, even when the same protein isoform is encoded (12, 13, 14). These noncoding regions are enriched in ultraconserved elements, regulatory regions with greater than 90% sequence identity for more than 100 bp that emerged more than 400 million years ago, before the evolution of lobe-finned fish and amphibians (15). Some promoters are ubiquitously active, whereas others are tissue, developmental time point, or disease-state specific (16, 17). Efforts to identify promoter-proximal proteomes to understand the molecular mechanisms governing alternative promoter selection require tools that can detect minute analytes across a wide dynamic range and specific targeting to resolve a ∼1 kbp promoter.

The isolation of sequence-defined chromatin regions represents a major biochemical challenge due to low intrinsic signal coupled to high background (18, 19, 20). There are only two counts of a single copy locus per diploid cell, and a 1 kbp promoter represents only ∼0.0001% of the Gbp sized human genome. Compounding the challenge is the biophysical similarity of chromatin—negatively charged DNA and positively charged nucleosomes with a large dynamic range of proteins of interest. The copy number of TFs per animal cell is on the order of 10,000 to 300,000 per nucleus (21), and there are tens of millions of histones per cell (22, 23). Early efforts to isolate sequence-defined chromatin regions focused on yeast to reduce background with its smaller genome (24, 25) or telomeres to increase signal due to their sequence abundance (26, 27). Recent efforts have utilized nuclease-dead dCas9-based strategies to target and isolate single copy loci in model organisms from human cells (28, 29) to plants (30, 31), typically relying on formaldehyde-crosslinking proteins to chromatin and isolating them. Complementary approaches have relied on proximity labeling enzymes like BioID or the engineered peroxidase APEX2 that can biotinylate proteins nearby their targeted region (32, 33). Several examples using dCas9-APEX2 demonstrate specific biotinylation of proteins physically near a targeted promoter region (34, 35, 36, 37, 38, 39, 40, 41, 42, 43, 44). These proximity labeling approaches preclude the need to cross-link chromatin, thereby enabling capture of chromatin-associated proteins that are not covalently linked to DNA by formaldehyde and allow for stringent wash steps using streptavidin-based purification of the labeled local proteome (45).

Mass spectrometry proteomics is the analytical tool often used for proteomic profiling of chromatin (46). While some approaches have compared the whole chromatin proteome between cell states (47, 48), advances in protocols and instrumentation have enabled deep proteome coverage even on material-limited samples including single cells (14, 49, 50) and subcellular spatial proteomes with organellar resolution (51, 52). However, these analyses do not uncover the proteins operating at single copy loci. Highly accurate quantitation and multiplexing can be achieved with tandem mass tags (TMTs) that barcode individual samples before combining them together for LC-MS analysis (53). A typical “bottom-up” data-dependent acquisition shotgun proteomics approach digests the protein fraction of samples to the corresponding peptides which are separated by liquid chromatography, measured on the mass spectrometer, and fragmented to produce characteristic patterns that are also measured on a mass analyzer (MS2) (54, 55). Extensive fractionation using an online 2D-LC system resolves complex chromatography peaks to allow for deeper proteome coverage (56). A third mass spectrometry step (MS3) to measure TMT signals ensures greater fidelity to the underlying ground truth quantitation by accounting for reporter ion ratio compression, and real-time database searching and synchronous precursor selection (RTS-SPS) economizes on instrument time and increases signal, respectively (57, 58, 59). The Orbitrap Ascend tribrid mass spectrometer design allows for deep proteome coverage using a TMT-based RTS-SPS-MS3 proteomics experiment (60, 61).

The TF *FOXP2* is among the most highly conserved genes across vertebrates in terms of sequence and location and developmental time of expression (62, 63, 64, 65). Originally disclosed as the first gene identified to be linked to Mendelian speech disorders, it has drawn a great deal of attention as a model to study evolution across vertebrates broadly and related to speech and vocalization in particular (66, 67, 68, 69, 70, 71, 72, 73, 74). The *FOXP2* locus harbors a cluster of 27 ultraconserved regions shared across vertebrates but not invertebrates, with only a dozen genes in the entire genome having a higher number of such conserved clusters (75). While some research has sought out downstream targets of *FOXP2* (76, 77, 78) or identified its role in developing brain (79, 80, 81), lung (82), and other tissues (83, 84), there have been fewer reports on regulation of *FOXP2* expression itself (85, 86). This may be partially explained by the complex patterns of regulation and expression: at least four separate alternative promoters can be active depending on the system under study, and further regulation can occur in *cis* from genomic elements 3 Mbp away from the upstream promoter (87). Understanding the molecular factors contributing to this complex regulation has relevance to human health as altered FOXP2 expression, both upregulation (88, 89) and downregulation (90, 91, 92), has been tied to various types of cancer and poor patient prognosis (93). The high degree of conservation *FOXP2* shows in its transcriptional regulation, coupled to its phenotypic relevance in vertebrate development and human health, make it an ideal model system to study the principles governing alternative promoter selection.

In an effort to unravel the highly conserved *FOXP2* regulatory module, we sought to deeply characterize in live HEK293 cells the proteins interacting with the active, upstream promoter of this single-copy locus (TSS1). We used genetically targeted proximity labeling with dCas9-APEX2 to covalently tag the promoter-proximal proteome with biotin. After streptavidin magnetic bead–based purification with stringent washing and on-bead digestion, we utilized online 2D-LC deep fractionation with TMT-enabled quantitative proteomics *via* RTS-SPS-MS3 on a tribrid mass spectrometer. Bioinformatic analysis on the 6029 proteins inferred by Proteome Discoverer (94) identified 373 proteins significantly enriched at the *FOXP2* promoter compared with the untargeted no gRNA control [Storey-*q* <0.05, fold change (FC) > 1.2]. This set of proteins was significantly enriched for TFs and spliceosome components. To benchmark our candidate proteins and provide a comprehensive characterization of potential regulators of the upstream promoter of *FOXP2*, we compared our proteomic results to multiple orthogonal approaches, including computationally predicted TFs recognizing the targeted region and the 223 Encyclopedia of DNA Elements (ENCODE) chromatin immunoprecipitation (ChIP)-Seq experiments performed in HEK293(T) cells (218 TFs, 5 histone modifications) (95, 96, 97, 98). We discovered and verified dozens of candidate regulators of the chromatin region at the active promoter of *FOXP2*. We also show that IRF2BP2 and glucocorticoid signaling each negatively regulate *FOXP2* expression in siRNA experiments and treatment with dexamethasone, respectively. We further demonstrate a uniform MS detection rate of approximately one third for binders and nonbinders at on target and off target loci, with more highly expressed genes significantly more likely to be detected by MS regardless of binding status or locus specificity. Proteins not detected by MS show significantly lower expression for binders *versus* nonbinders only at the on target TSS1, while there is no significant expression difference for undetected proteins at off target loci.

## Experimental Procedures

### Experimental Design and Statistical Rationale

For proteomics experiments, we employed two negative controls: one without labeling occurring to account for endogenously biotinylated proteins and nonspecific interaction with streptavidin magnetic beads (NoG-) and one with untargeted labeling to account for proximity labeling background from dCas9-APEX2 molecules not associating with targeted locus within the cell (NoG+). To increase signal of the promoter proximal proteome, we tiled the locus with three gRNAs in separate cell lines as pseudo-technical replicates. To account for biological and technical batch-based variation, we required a minimum of three biological replicates, totaling 15 separate conditions for analysis. These requirements are achievable with TMTpro which is capable of 18-plexing. Each of the five conditions were processed in parallel for each biological replicate until all samples were combined after TMT labeling. For ChIP, each of the four conditions (untargeted NoG negative control plus three on target sgRNAs) had three biological replicates. Each biological replicate produced two technical replicates for ChIP for a total of n = 6 per condition. As with proteomics experiments, the targeted sgRNA cell lines were pooled together as pseudo technical replicates, and conditions within a biological replicate were processed simultaneously. For protein bioinformatic analysis, we report adjusted *p*-values to account for the multiple hypothesis comparison problem. When filtering out background, we used strict Bonferroni correction to limit false positives being inappropriately called contaminants (99). When testing for enrichment, we used Storey correction to be more permissive in calling hits (100, 101). Global quantification data approximated a negative binomial distribution before log_2_ transformation, after which it approximated a normal distribution. To test statistical significance of protein types in enriched *versus* total sample, we performed a χ^2^ test on the categorical data (in class/not in class). We performed Cochran’s Q test to ascertain whether the proportion of ENCODE TFs binding at analyzed loci significantly differed. We used McNemar’s test for pairwise comparisons of individual loci as a follow up to Cochran’s Q test with Bonferroni correction to call significance.

### Cell Line Construction and Culture

HEK cells were cultured in Dulbecco’s Modified Eagle Medium containing 10% FBS and 1% penicillin–streptomycin and passaged every 3 days. Cells were maintained at 37 °C in a humidified atmosphere containing 5% CO_2_. To stably incorporate the Caspex construct, cells were transfected with Lipofectamine 3000 (Invitrogen) according to the manufacturer’s instructions and selected for 2 weeks with 4 μg/ml puromycin in the media. Inducible Caspex expression was a gift from Steven Carr & Samuel Myers (Addgene plasmid #97421) (38). Single colonies were selected by fluorescence activated cell sorting (FACS) at the Stanford Shared FACS Facility (SF1a) and tested for doxycycline inducibility of Caspex monitored by GFP visualization and anti-FLAG Western blotting with Rabbit M2 (Sigma F2555). Individual sgRNA constructs were transfected into the best responding Caspex cell line and selected with 200 μg/ml hygromycin. Cell lines were maintained with 4 μg/ml puromycin and/or 200 μg/ml hygromycin to maintain selection pressure for the Caspex and sgRNA plasmids, respectively, until use in ChIP-PCR or proximity labeling experiments.

### Plasmid Construction

The UCSC Genome browser was utilized to select sgRNA sequences targeting the promoter of *FOXP2* that conformed to requirements for expression from the U6 promoter while minimizing off target effects. Selected guides were required to start with G for transcription initiation and were rejected if they contained a run of 4 or more T’s in a row to prevent early termination. The MIT specificity score for all guides was greater than 90. Appropriate gRNA sequences were cloned into the lenti-sgRNA hygro backbone, a gift from Brett Stringer (Addgene plasmid #104991) (102). Briefly, the lenti-sgRNA hygro plasmid was digested with *BsmBI* (New England Biolabs) to create overhangs that could hybridize with Fwd-5′-ACACCGN_20_G-3′ and Rev-5′-AAAACN_20_G-3′ sequences, which were ligated with the designed oligonucleotides using T4 polymerase (New England Biolabs). The vector was transformed into One Shot Stbl3 chemically competent *E. coli* (Invitrogen), single colonies were picked, and plasmid DNA was purified with a QIAGEN EndoFree Maxiprep Kit. Successful gRNA incorporation was confirmed by Sanger sequencing (Integrated DNA Technologies). Primers for gRNA incorporation were synthesized by Integrated DNA Technologies and were as follows: g1 Fwd-5′-ACACCGCAGACACCTTTCGGTGATAG-3′, Rev-5′-AAAACTATCACCGAAAGGTGTCTGCG-3′; g2 Fwd-5′-ACACCGACACCTTTCGGTGATAGGGG-3′, Rev-5′-AAAACCCCTATCACCGAAAGGTGTCG-3′; g3 Fwd-5′-ACACCGTTATCCCGAAGCGTCAGTAG-3′, Rev-5′-AAAACTACTGACGCTTCGGGATAACG-3′ to target sequences in chr7 (hg38) of g1:114087491-114087513, g2:114087494-114087516, g3:114087279-114087301. Target sequences were chosen for proximity to the TSS and overlapping labeling radii (∼400 bp (38)) within the *FOXP2* TSS1 promoter while conforming to U6 expression requirements.

### Cross-Linking and Sonication for ChIP

Doxycycline (70% in ethanol) was added to cells at a final concentration of 1 μg/ml in a 15-cm^2^ plate for 21 h so Caspex expression would be induced when cells were approximately 90% confluent (∼10^7^ cells). Caspex expression was visually confirmed by fluorescence of the GFP marker. A single cell suspension was crosslinked with 1% formaldehyde at room temperature with rotation for 10 min. To quench formaldehyde, 2M glycine was added to a final concentration of 125 mM and incubated for 5 min at room temperature with rotation. Cells were washed twice with ice-cold PBS, pelleted, snap-frozen in liquid nitrogen, and stored at −80 °C until use. Cells were thawed at 4 °C in ice-cold PBS with rotation for 30 min. Pelleted cells were treated with 3 ml hypotonic buffer (20 mM Hepes pH 7.9, 10 mM KCl, 1 mM EDTA pH 8.0, 10% glycerol) with protease inhibitors [Roche cOmplete protease inhibitor (1 tablet/50 ml), 0.5 mM PMSF] and 1 mM DTT added just before use. The plasma membrane was allowed to swell for 10 min before shearing with 30 strokes of a Dounce homogenizer. Total time for swelling and homogenization was limited to 15 min. Nuclei were pelleted and washed once with ice-cold hypotonic buffer before lysis in 3 ml ice-cold radioimmunoprecipitation (RIPA) buffer (150 mM NaCl, 50 mM Tris-HCl pH 8.0, 1% IGEPAL CA-630, 0.5% sodium deoxycholate, and 0.1% SDS) with protease inhibitors, DTT, and phosphatase inhibitors (1 mM Na_2_P_2_O_4_, 2 mM Na_3_VO_4_, 10 mM NaF) added just before use. Lysed nuclear pellets were sonicated on ice to shear chromatin for solubilization with a SFX250 Sonifier (Branson) set to an intensity (output control) of 3.5. Lysates were sonicated for 16 rounds of 30 s on and 30 s off, taking care to prevent foaming. To prevent sample overheating, lysate was allowed to rest on ice for at least 2 min every four cycles. Lysate was clarified by centrifugation at 14,000 rpm for 15 min at 4 °C. Supernatant was transferred to a 15 ml Falcon tube, diluted to 4 ml total in RIPA buffer, flash frozen in liquid nitrogen, and stored at −80 °C until use. Chromatin fragmentation was assessed after de-crosslinking (*vide infra*) using a Bioanalyzer (Agilent). Typical results produced an average size of ∼530 bp with 55 to 60% of fragments in the 200-1000 bp range (SF1b).

### Chromatin Immunoprecipitation

For chromatin immunoprecipitation, each biological replicate produced two technical replicates for pulldown. For one technical ChIP replicate, 2 ml of sonicated lysate was used. Before pulldown, 20 μl input (1%) was removed to compare nucleic acid enrichment before and after ChIP. Sonicated lysate was incubated at 4 °C overnight with 5 μg FLAG Rabbit M2 monoclonal antibody (Sigma F2555) with rotation. A 1:1 mixture of 150 μl total Protein A:Protein G magnetic beads (Invitrogen Dynabeads, 30 mg/ml each) was washed twice with 1 ml ice-cold RIPA buffer to preclear the beads. The beads were transferred fully to the antibody-complex-chromatin mixture and incubated for 1 h at 4 °C with rotation. The beads were washed three times with ice-cold RIPA buffer with inhibitors added followed by a wash with ice-cold PBS. The beads were transferred from the 15 ml Falcon tube to a 2 ml DNA lo-bind Eppendorf tube with 800 μl + 2 times 400 μl ice-cold PBS to ensure complete transfer. The PBS was removed, and beads were incubated with 100 μl of freshly made 1% SDS, 10 mM Tris pH 8.0, and 1 mM EDTA at 65 °C for 10 min with gentle mixing by vortex every 2 min. The supernatant was collected, and beads were incubated with 150 μl of 0.67% SDS, 10 mM Tris pH 8.0, and 1 mM EDTA at 65 °C for 10 min with gentle mixing every 2 min before combining both eluates. Input DNA (stored at 4 °C overnight) was diluted 1.5x in 1% SDS, 10 mM Tris pH 8.0, and 1 mM EDTA before both ChIP DNA and input DNA had crosslinks reversed overnight at 65 °C. An equal volume of 1% SDS, 10 mM Tris pH 8.0, and 1 mM EDTA with 100 μg RNase A (QIAGEN) was added to de-crosslinked DNA and incubated for 30 min at 37 °C followed by addition of 5.0 μl of 20 mg/ml Proteinase K (QIAGEN) and incubation at 45 °C for 30 min to remove RNA and proteins. DNA was purified with QIAGEN QIAquick purification columns and used for qPCR. De-crosslinked and purified input DNA (1 μl) was used for the Bioanalyzer assay to assess chromatin fragmentation.

### qPCR

Each pulldown was analyzed in quadruplicate using SYBR Green qPCR (Applied Biosystems, A46012). Input DNA was typically diluted 10× to reach similar concentration to ChIP DNA. Forward and reverse primers synthesized by Integrated DNA Technologies at 400 nM were combined with 2 μl DNA and 10 μl SYBR Green Mix in a 20 μl final reaction volume. Enzyme was activated at 95 °C for 2 min before 40 cycles of denaturation and annealing (95 °C for 5 s, 60 ˚C for 30 s). The following primers were used (proximity of g1 and g2 allowed the same primer pair): g1/g2 Fwd-5′-TGGCTGTTTGTGGGTGGGTTT-3′, Rev-5′-GAAGCCCTCCCTATCACCGAA-3′; g3 Fwd-5′-GGAGTCAAGAAACTCCTGGGC-3′, Rev-5′-TCAAGGCAGCAGTCATCCCT-3′; *GAPDH* control Fwd-5′-TTGGCTACAGCAAGAGGGTG-3′, Rev-5′-GGGGAGATTCAGTGTGGTGG-3′. Enrichment was determined using the 2^−ΔΔCt^ method (103). Input DNA was normalized to 100% by subtracting the appropriate dilution factor from the raw Ct value (*e.g.,* 9.966 for 10× dilution of 1% input [log_2_{1000}]). The Ct values for each individual gRNA from a single technical replicate were averaged. The degree of enrichment after ChIP was calculated by comparing the Ct of ChIP DNA for the condition of interest normalized to the off-target *GAPDH* control with the Ct of adjusted input DNA normalized to off target *GAPDH*, *i.e*., ChIP(Ct[pooled gRNAs or NoG] – Ct[*GAPDH*]) – adjusted input(Ct[pooled gRNAs or NoG] – Ct[*GAPDH*]) = ΔΔCt. The FC was calculated as 2^−ΔΔCt^. FC for biological and technical replicates were averaged and presented numerically as mean ± SEM and visually as a box and whisker plot.

### Proximity Labeling

Cells were treated with 1 μg/ml doxycycline 21 h before labeling, and Caspex expression was confirmed by fluorescence of the GFP marker. Biotin tyramide phenol (APExBIO) was diluted in external media from a 500 mM stock solution in DMSO to solubilize before being added to cells at a final concentration of 500 μM. After at least 30 min of incubation to allow time for the rate-limiting step of biotin diffusion across membranes (104), hydrogen peroxide was diluted in media to 100 mM before addition to the cells at a final concentration of 1 mM to induce biotinylation. After 60 s of very gentle swirling, the media were decanted as quickly as possible, and cells were washed three times with ice-cold quenching solution (100 mM sodium azide and 100 mM sodium ascorbate in PBS), taking care to avoid dislodging the cells from the dish. Cells were washed with once ice-cold PBS to help remove excess biotin phenol, transferred to 15 ml Falcon tubes, pelleted, flash frozen in liquid nitrogen, and stored at −80 °C until use. For confirmation of proximity labeling, one 10-cm^2^ dish was used per biological replicate. For mass spectrometry studies, eight 15-cm^2^ dishes were used per replicate per condition.

### Western Blot

Labeled cell pellets were thawed at 4 °C with gentle rotation in ice-cold PBS. Pelleted cells were lysed in ice-cold RIPA with freshly added protease and phosphatase inhibitors, DTT, and 10 mM sodium ascorbate and 10 mM sodium azide added to inhibit APEX2 to prevent excess labeling with any remaining adventitious biotin phenol. Lysate was sonicated on ice for three 10 s cycles. Forty micrograms of protein were denatured in NuPAGE LDS sample buffer (Invitrogen) supplemented with 50 mM DTT, followed by SDS-PAGE using 4 to 12% Bis-Tris WedgeWell Gel (Invitrogen) in MOPS running buffer (Invitrogen). Proteins were transferred to nitrocellulose membranes (0.2 μm, Bio-Rad) with a Trans-Blot Turbo Transfer System (Bio-Rad), blocked with 5% milk in TBST (30 min), and incubated with GAPDH Rabbit monoclonal RM114 antibody (Sigma SAB5600208) at 4 °C overnight at a 1:2000 dilution. Following primary antibody incubation, membranes were washed three times with TBST, incubated with horseradish peroxidase-conjugated secondary anti-rabbit IgG (Cell Signaling 70745) and Streptavidin (Abcam AB279315) for 30 min (1:1000 and 1:10000 dilutions, respectively). Membranes were washed four times, and signals were developed with chemiluminescence and imaged using the ChemiDoc Imaging system (Bio-Rad). Images were processed with the Fiji implementation of ImageJ (105). Changes in contrast were applied to the entire blot.

### Enrichment of Biotinylated Proteins and on Bead Digestion

Cell pellets were thawed at 4 °C with gentle rotation in cold PBS. Pelleted cells were lysed in 3 ml ice-cold RIPA buffer with protease and phosphatase inhibitors, DTT, sodium ascorbate, and sodium azide added fresh before use. Lysate was sonicated as described above for ChIP. Typical sonication conditions resulted in an average size of ∼450 bp with 85 to 90% of chromatin between 200 and 1000 bp as assessed by the Bioanalyzer assay (SF1c). Protein concentration was determined by the Bradford assay at 595 nm (Abcam AB102535)—the addition of redox quenchers to prevent excess biotin labeling precludes use of the common BCA assay. For each condition, 500 μl of a Streptavidin M280 magnetic bead slurry (Dynabeads Invitrogen, 10 mg/ml) was utilized. The magnetic beads were washed twice with ice-cold RIPA buffer. Lysates of equal protein amounts for each condition were incubated with precleared beads for 120 min at room temperature. After capture of biotinylated proteins in the Falcon tube, beads were fully transferred to a 2 ml Eppendorf protein lo-bind tube. Contaminants were removed by washing twice with 1 ml lysis buffer, once with 1M KCl, once with 100 mM Na_2_CO_3_, and twice with 6M guanidinium chloride (GdnCl) in 50 mM triethylammonium bicarbonate (TEAB) (all ice-cold). Proteins were denatured, reduced, and alkylated by incubation with 100 μl 6M GdnCl, 10 mM TCEP, 40 mM chloroacetamide in 100 mM Tris pH 8.5 for 5 min at 95 °C followed by 55 min at room temperature. Beads were washed four times with ice cold 50 mM TEAB to remove excess GdnCl to prevent incomplete protease digestion. 1% of the bead mixture was removed to check capture efficiency *via* biotin elution (*vide infra*) before digestion. To a 20 μl slurry of bead-bound proteins in TEAB was added 400 ng (2 μl) of trypsin/LysC (Promega) (106), and the reaction was incubated at 37 °C overnight. Digestion was quenched by the addition of 10% trifluoroacetic acid (TFA) to a final concentration of 1%. Supernatant was collected, and the beads were washed with 25 μl of 1% TFA, and supernatants were combined. Peptides were stored at −80 °C until use. Remaining beads were eluted with biotin buffer to check digestion efficiency (45). Briefly, beads were incubated with 50 μl 2x LDS loading buffer, 200 mM DTT, and 15 mM biotin for 15 min at 95 °C two times. Combined supernatants were diluted 2x in LDS loading buffer and used for Western blot.

### TMT Labeling

Peptides were desalted with a Waters Oasis HLB Cartridge before labeling with TMTpro 18-plex reagent (Thermo) according to the manufacturer’s protocol. Briefly, TMT label reagents in anhydrous acetonitrile were added to each sample. The TMT isotopic labels were randomized across the different conditions according to the following scheme: g2 Rep1:126; g2 Rep2:127N; g1 Rep3:127C; g1 Rep1:128N; NoG + Rep3:128C; NoG + Rep1:129N; NoG- Rep1:129C; empty:130N; NoG + Rep2:130C; NoG- Rep2:131N; g3 Rep3:131C; g3 Rep1:132N; g3 Rep2:132C; sample pool: 133N; g1 Rep2:133C; NoG- Rep3:134N; g2 Rep3:134C; empty:135N, where Rep# refers to the biological replicate. The labeling reaction was allowed to proceed for 1 h at room temperature before quenching with 5% hydroxylamine for 15 min. Samples were dried with a SpeedVac (Thermo) before resuspension in 100 mM ammonium formate for LC-MS analysis.

### Online 2D-LC and Data Acquisition via RTS-SPS-MS3

Data were collected with a Waters Acquity UPLC M-Class 2D-LC system directly connected to an Ascend Tribrid Mass Spectrometer (Thermo). A 12 multifraction gradient over 220 min was utilized with injection of 15 μg of peptides. Peptide amount was estimated using calibration runs on the MS with equal volume of each TMT-labeled sample injected to ensure equal peptide loading in the data acquisition run. The first dimension of separation at high pH across a BEH column consisted of buffer A (20 mM ammonium formate at pH 10) and buffer B (acetonitrile) using 12 discontinuous steps of buffer B at 10.8%, 13.1%, 14.9%, 16.7%, 17.7%, 18.9%, 19.9%, 20.4%, 22.2%, 25.8%, 28.9%, and 45% at a flow rate of 2 μl/min before transfer to a low pH C18 analytical column (75 μm ID/10 μM tip ID x 28 cm C18-AQ 1.8 μm resin). For each step, a 5 min isogradient of %B was used (SF2). TICs in SF2 were produced with the RaMS package in R (107). In the second dimension, a linear gradient from 5% to 30% buffer B (0.1% formic acid in acetonitrile) in buffer A (0.1% formic acid in water) at a flow rate of 300 nl/min was used and directly injected to the mass spectrometer. The 3s duty cycle scan sequence began with an Orbitrap MS1 spectrum with the following parameters: resolution 120,000, scan range 400 to 1500 *m/z*, automatic gain control (AGC) target of 400,000 (100%), maximum injection time 50 ms, and RF lens 50% with a minimum of 6 points desired across the peak. Precursors were filtered using monoisotopic peak determination, charge state 2 to 7, and dynamic exclusion of 60 s with a ±10 ppm tolerance excluding isotopes and different charge states. MS2 spectra were collected in the linear ion trap at a rapid scan rate with precursor fit of 50% in a 0.7 *m/z* window with a scan range of 400 to 1500 *m/z* (*mode auto*). Ions were fragmented with collision induced dissociation (CID) at a collision energy of 33% (activation time 10 ms, Q = 0.25) with a maximum injection time of 40 ms and AGC target of 25,000 (250%). MS2 ions were selected by quadrupole across a 300 to 1500 *m/z* range for RTS-SPS analysis against the UniProt 2022 human protein database. A maximum of four peptides per protein were selected for RTS-SPS analysis. Enzyme specificity was set to Trypsin/P with a maximum of one missed cleavage and 35 ms maximum search time. A maximum of two variable modifications were allowed. Static modifications of cystine carbamidomethylation (57.0215 Da) and lysine and n-terminal TMTpro modification (304.2071 Da) plus variable methionine oxidation (15.9949 Da) were included for selection to MS3. Scoring thresholds for each charge state were set to Xcorr = 2.5(2+), 2.6(3+), 3.2(4+), and 3.2(5+) with dCn = 0.1 for all states. MS3 was measured in the Orbitrap at a resolution of 60,000 with fragmentation achieved *via* higher-energy collisional dissociation (HCD) at 65% collision energy. The scan range consisted of 100 to 500 *m/z* with a maximum injection time of 150 ms, AGC target of 100,000 (200%), and eight precursors being isolated.

### Protein Inference

Protein groups consistent with identified peptides were inferred with Proteome Discoverer 2.5 (Thermo). Raw files were searched against the UniProt 2022 human proteome database consisting of 20,324 sequences. Mass tolerance of 10 ppm was used for precursor ions and 0.6 Da for fragment ions. The search included cysteine carbamidomethylation (57.0215 Da) as a static modification. Peptide N-terminal and lysine TMTpro 18plex modification (304.2071 Da), protein N-terminal acetylation (42.0106 Da), and methionine oxidation (15.9949 Da) were set as dynamic modifications. Up to two missed cleavages were allowed for trypsin digestion. The peptide false discovery rate (FDR) was set as <1% using Percolator (108) with a reversed-sequence decoy database (109). Only peptide level FDR was applied in the search parameters. For protein identification, at least one peptide with a minimum six amino acid length was required. A total of 339 decoys and 6029 target proteins were detected for a protein level FDR of 5.6%. Peptide spectral matches (PSMs), peptide groups, and inferred proteins resulting from Proteome Discoverer analysis are presented in supplemental Tables S1–S3, respectively. Grouped abundance for proteins from each TMT channel in supplemental Table S3 was used for bioinformatic analysis in Excel.

### Bioinformatic Analysis

Grouped abundance of proteins was first median normalized within each TMT channel, after which biological replicates were averaged across TMT channels. Individual gRNA cell lines were treated as pseudo-technical replicates and averaged together for analysis. Raw quantification values approximated a negative binomial distribution before and after median normalization and were log_2_ transformed to approximate a normal distribution. The difference between log_2_ quantified conditions of interest for individual proteins was converted to a Z-score to enforce normality for statistical analysis, and a corresponding two-tailed *p*-value was calculated. The Z-score was calculated as x/σ, where x = difference in log_2_(median normalized protein quantification) and σ = standard error of the mean. Quantification error of the SEM for the log_2_ transformation was propagated *via* the delta method (d/dx log_2_(σ) = σ∗(1/y∗ln(2)), where y = median normalized quantification). Error of summation was propagated in the standard way (square root of the sum of squared errors). Proteins that were not quantified were excluded from analysis (965 total). Proteins that were not detected across all four conditions (NoG-, NoG+, pooled gRNAs, and sample pool of all replicates) were excluded from analysis to prevent undefined ratios after manual inspection to assess potential biological significance, removing a total of 49 proteins. A contaminant list was created by comparing NoG- to the pooled labeling conditions (all gRNAs + NoG+) and proteins enriched by 1.2FC and Bonferroni adj. *p* < 0.05 were excluded from downstream analysis. Differences between pooled on-target conditions *versus* NoG+ from the resulting list were then analyzed. Proteins with a FC > 1.2 and a Storey-*q* value < 0.05 were considered enriched. Calculated *p*-values of zero were replaced with the lowest calculated *p*-value to prevent an undefined value in the volcano plot.

### Gene Ontology Analysis

Gene ontology analysis was performed with DAVID, GORilla, and Pantherdb. For each tool, the list of 6029 inferred proteins was uploaded as a background list rather than using the whole human proteome to test for GO enrichment. Proteins enriched by FC > 1.2 in on target *versus* NoG + conditions with (373) or without (775) the *q*-value filter were tested for enrichment of GO Biological Process, GO Cellular Component, and GO Molecular Function in each tool. For PantherdbGO, GO complete terms were searched, and Fisher’s exact test with Benjamini–Hochberg correction was used to determine significance. For DAVID, the top three Functional Annotation Clusters were recorded alongside the Functional Annotation Chart with Benjamini–Hochberg correction used for all comparisons. Benjamini–Hochberg correction was used for GORilla analysis in each category. Results are presented in supplemental Table S4.

### Predicted TF Binding

For TFBSPred, the first TSS of FOXP2 (hg 38:7:114086327(+) - 114087534) was chosen for analysis (accessed August 2024). Every box was checked for lineage, tissue, karyotype, and sex. Every cell line was chosen for analysis, and the default TFFM (TF Flexible Model) search threshold of 0.90 was used. Every predicted TF present within the whole sequence was compared against our mass spectrometry results. For PROMO, *homo sapiens* was selected for the species, and the same sequence analyzed by TFBSPred was used for input (accessed August 2024). All predicted TFs were recorded and compared to mass spectrometry data in supplemental Table S6.

### Comparison with ENCODE ChIP-Seq Database

All published TF and histone experiments that passed ENCODE data quality standards in the ChIP-Seq data matrix performed in HEK293 or HEK293T cells available at encodeproject.org were analyzed. The ENSCR experiment identifiers and associated laboratories for the 223 analyzed experiments are recorded in supplemental Table S7. To determine if a factor binds to the *FOXP2* genomic locus, the web-based genome browser tool for each experiment was utilized. Only signals that were called as significant by ENCODE in the combined replicate experiments that reached the irreproducible discovery rate threshold were considered as true hits—a significant signal in one or both individual replicates that did not persist in the combined experiments was discarded. Each experiment was analyzed at the following genomic loci (hg38 chr7): TSS1: 114084641-114087534; e330: 114411165-114418591; upstream intergenic region: 114075920-114084640; *FOXP2* gene body: 114086327-114693773. Any peaks that passed the irreproducible discovery rate threshold within the analyzed region were recorded as a binary Yes/No in supplemental Table S7. Multiple significant peaks within a defined analyzed region were individually counted for the n > 3 condition. Locus specificity of binding was determined by analyzing off target regions at the following genomic loci: *GAPDH* (hg38 chr12): 6533595-6536613; *IRX6* (hg38 chr16): 55322957-55326110; *MAGI1* (hg38 chr3): 66037224-66040189; and *NANOG* (hg38 chr12): 7787127-7789975.

### Statistical Analysis

To assess statistical significance of enrichment values for ChIP-qPCR, expression changes for siRNA, and mean expression for MS detected *versus* not detected TFs, a two-sided *t* test was performed. To determine enrichment of TFs and components of the spliceosome, the χ^2^ test was performed on pairwise comparisons after creating a contingency table. For protein quantification, the two-tailed *p*-value corresponding to the Z-score calculated for difference in protein quantification between states of interest was calculated. To correct for multiple hypothesis testing across the thousands of quantified proteins, Bonferroni or Storey methods were used as indicated in the text. For Bonferroni correction, the significance threshold was set at α/n, where α = uncorrected significance threshold (set to 0.05) and n = number of comparisons. Storey correction is a modification of Benjamini–Hochberg correction (110) where the proportion of null hypotheses is estimated to be less conservative in calling significance. The results from the n statistical tests were listed in ascending order of *p*-value and assigned a rank order of 1,2,…,n. The proportion of null hypotheses, π_0_, was estimated according to the formula π_0_ = (#*p*-values>λ)/(n∗[1-λ]), where λ = 0.4 as estimated from the flat portion of the *p*-value histogram. The value λ represents uniform distribution of truly null values considering the fact that truly significant values will skew toward lower *p*-values. The *q*-value for element *i* was calculated as the lower of (π_0_∗n∗*p*-value_*i*_)/rank_*i*_ and *q*-value(*i* + 1). Cochran’s Q test was calculated according to the formula (k∗[k-1])∗(Σ[{column total - N/k}ˆ2]/Σ[row total∗{k - row total}]), where k = number of loci and N = grand total, with genomic loci in columns, TFs in rows, ENCODE positive = 1, and ENCODE negative = 0 and compared to the χ^2^ distribution. McNemar’s test on pairwise comparisons was calculated according to the formula (b-c)^2^/(b + c), where b and c are discordant entries in the contingency table (1Y2N and 1N2Y) and compared to the χ^2^ distribution with Bonferroni correction.

## Results

### Preparing the FOXP2 Promoter-Proximal Proteome for Mass Spectrometric Analysis by Genetically Targeted Proximity Labeling

**Fig. 1:**
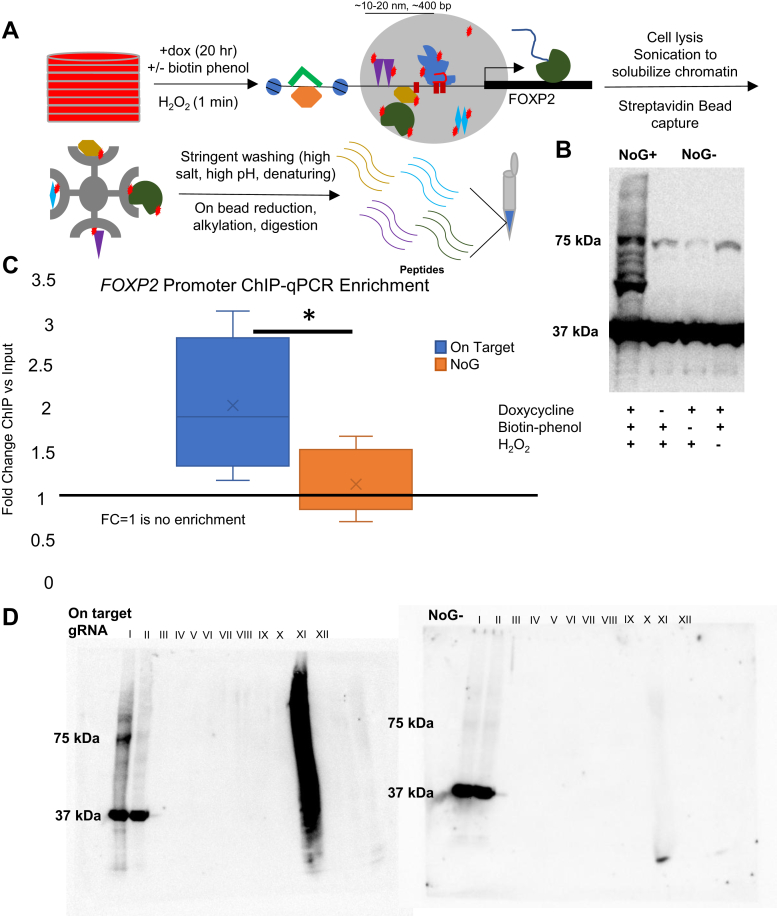
**Preparing the *FOXP2* promoter-proximal proteome for mass spectrometric analysis by genetically targeted proximity labeling.***A,* schematic overview of experimental approach for promoter-pulldown proteomics. A large number of input cells (8 15-cm^2^ plates per condition per replicate) are used to generate sufficient material for MS analysis. Activation of genetically targeted proximity labeling by dCas9-APEX2 creates a cloud of reactive phenoxyl radicals (*gray circle*) that covalently biotinylate proteins (*red stars*) within 10 to 20 nm of the promoter targeted by the designed sgRNAs (designated by *red boxes*). Sonication to solubilize chromatin enables capture of biotinylated proteins by streptavidin magnetic beads. Stringent washing removes background contaminants before on-bead digestion for mass spectrometric analysis of generated peptides. *B*, Western blot showing proximity labeling positive and negative controls. All samples show the GAPDH loading control and endogenous 75 kDa biotinylated proteins. Systematic exclusion of necessary chemicals prevents proximity labeling, visible as the smear in the condition with all reagents included. The condition with all labeling reagents and no gRNA (NoG+) and the condition with exclusion of biotin phenol (NoG-) are representative of negative controls in the MS experiment. *C*, enrichment of targeted FOXP2 promoter using ChIP-qPCR by the 2^-ΔΔ^^Ct^ method. The region targeted by the sgRNAs was enriched by 2.06 ± 0.31, while the untargeted NoG + condition showed no enrichment (1.15 ± 0.15) compared to an off-target *GAPDH* control. ∗*p* < 0.05, n = 6. *D,* Western blot demonstrating capture and retention of biotinylated proteins during stringent washing for targeted labeling (gRNA2, *left*) and untargeted no labeling (NoG-, *right*) conditions. GAPDH loading control is visible in both conditions, while biotin tagging visualized by Streptavidin-HRP is only present in labeling positive conditions. The biotinylated proteins from input are efficiently captured by the streptavidin beads and show no elution during wash steps. 1% of beads predigestion demonstrates labeled proteins remain captured, while the absence of signal in the final lane indicates on-bead digestion went to completion. I: input; II: flow-through; III: RIPA wash 1; IV: RIPA wash 2; V: 1M KCl wash; VI: 100 mM Na_2_CO_3_ wash; VII: 6M GdnCl wash; VIII: denaturation, reduction, alkylation; IX: TEAB wash 1; X TEAB wash 4; XI: 1% bead elution predigestion; XII: remaining bead elution postdigestion. ChIP, chromatin immunoprecipitation.

The experimental approach is outlined in Figure 1*A* and used a modified protocol. We began our investigation by generating stable HEK293 cells expressing dox-inducible dCas9-APEX2. Only a small fraction of cells responded to doxycycline treatment after puromycin selection, so we used FACS to generate a clonal population to minimize background signal from unlabeled chromatin in nonexpressing cells for downstream experiments (supplemental Fig. S1*A*). After confirming competence in proximity labeling (Fig. 1*B*), we generated three stable cell lines each expressing a different gRNA within the active *FOXP2* promoter region. We performed ChIP-qPCR against the FLAG tag on the proximity labeling construct to confirm accurate targeting using the 2^−ΔΔCt^ approach to compare enrichment of targeted *versus* untargeted conditions relative to a background locus (103). The no gRNA negative control showed no enrichment after ChIP compared to an off target *GAPDH* control locus (FC = 1.15 ± 0.15, where FC = 1 is no change) (Fig. 1*C*). By contrast, the pooled on-target gRNAs targeting the *FOXP2* promoter showed significant enrichment after ChIP (FC = 2.06 ± 0.31, *p* < 0.05) (mean ± SEM, n = 6). This degree of enrichment is consistent with other locus-specific chromatin isolation qPCR results (40, 43, 44). We anticipate this to represent a lower bound of enrichment given that streptavidin-based purification can enrich on-target loci by up to 284-fold when compared head-to-head with FLAG-based enrichment (38, 111).

After confirming successful targeting to the *FOXP2* promoter, we covalently tagged the proximal proteome with biotin using standard proximity labeling conditions in which 1 min of H_2_O_2_ treatment catalyzed biotinylation of surface exposed tyrosine residues within an approximately 20 nm radius. We sonicated chromatin to an average size of ∼450 bp to solubilize labeled chromatin proteins (supplemental Fig. S1, *B* and *C*) and captured biotinylated proteins with streptavidin magnetic beads. To reduce capture of nonspecific background, we increased the stringency of wash steps, replacing the standard 2M urea wash with 6M GdnCl. Proteins adopt a random coil conformation at 6M GdnCl, whereas the midpoint of unfolding is at 3M urea (112). Given that the half time for biotin-bound streptavidin unfolding in 6M GdnCl is 50 days (113), we hypothesized a wash with 6M GdnCl would remove more contaminants while maintaining capture of biotinylated proteins. Accordingly, we observed no loss of material eluting in flow through by Western blot before the final step (Fig. 1D). We performed on-bead denaturation, reduction, alkylation, and digestion to generate peptides for mass spectrometric analysis.

### Extensive Online Fractionation and Quantitative Proteomics via 2D-LC-TMT-RTS-SPS-MS3

**Fig. 2:**
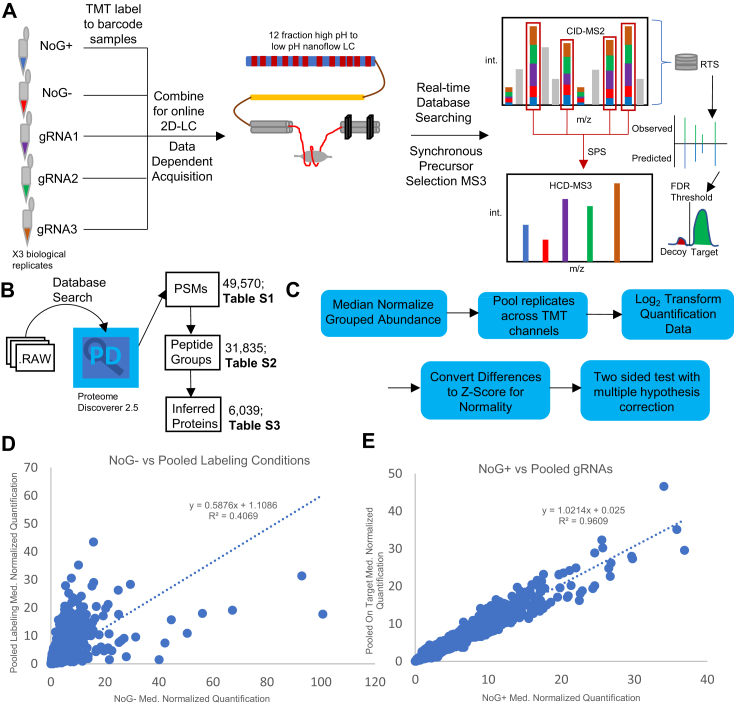
**Extensive online fractionation and quantitative proteomics *via* 2D-LC-TMT-RTS-SPS-MS3.***A*, overview of 2D-LC-TMT-RTS-SPS-MS3 mass spectrometry workflow. Three biological replicates representing five conditions (three on target sgRNAs, plus two negative controls) are barcoded with TMTs before combination and injection on the LC-MS. A high pH 12-fraction gradient followed by an analytical low pH column separates peptides by retention time before direct connection to the tribrid MS instrument. A quadrupole selects ions in a defined *m/z* range for a high resolution orbitrap scan to capture MS1. Ions are sent to the linear ion trap for MS2 and eventually return to the orbitrap for MS3 quantification. Fragments in MS2 are compared in real-time on the instrument to a protein database to ensure only signals that can be matched to peptides with high confidence are sent to MS3 to economize on the instrument duty cycle, and multiple precursors are selected. Peptide-level FDR is controlled with a decoy database strategy. *B*, raw files from the mass spectrometer were processed using a database search *via* Proteome Discoverer. Total number of identified PSMs (49,570; supplemental Table S1), peptide groups (31,835; supplemental Table S2), and inferred proteins (6029; supplemental Table S3) are indicated. *C,* bioinformatic workflow to analyze protein quantification. *D,* global pairwise protein quantification comparing NoG-against all conditions in which labeling occurred (pooled gRNAs + NoG+). There is low correlation in protein quantification (R^2^ = 0.41) between NoG- and conditions where labeling occurred. *E,* global pairwise protein quantification comparing NoG + against pooled on target conditions. There is a high degree of correlation in protein quantification (R^2^ = 0.96) between on target and untargeted conditions. FDR, false discovery rate; MS3, third mass spectrometry step; PSM, peptide spectral match; RTS, real-time database search; SPS, synchronous precursor selection; TMT, tandem mass tag.

The full mass spectrometry data acquisition workflow is outlined in Figure 2*A*. In addition to the three individual gRNA cell lines tiling the *FOXP2* promoter, we included two negative controls: no gRNA with exclusion of biotin phenol to account for endogenously biotinylated proteins and nonspecific bead interactors to generate a high confidence contaminant list (NoG-) and a no gRNA condition with biotin phenol to control for background labeling (NoG+) (see Fig. 1*B*). Each condition was comprised of eight 15-cm^2^ plates and had three independent biological replicates, comprising more than a billion cells (10^9^, although only 60% represent on target chromatin). The five conditions for each biological replicate were processed in parallel. The 15 conditions plus a pool of equal amounts of each sample were combined together after labeling with TMTs for a 16-plex experiment, and 15 μg of protein was injected for LC-MS.

In order to reduce the signal loss from repeated sample handling steps (114) while maintaining the benefits of deep proteome coverage from extensive fractionation, we utilized online 2D-LC directly connected to the mass spectrometer. A 12-fraction high-pH gradient separated peptides before transfer to a low pH nanoflow column (supplemental Fig. S2). Using RTS-SPS-MS3 for quantitative proteomics (see methods), we detected 49,570 PSMs *via* database search (Supplemental Table S1). We utilized Proteome Discoverer to infer proteins consistent with the 31,835 identified peptide groups (Supplemental Table S2), generating a list of 6029 proteins (Fig. 2*B*) (Supplemental Table S3). Since this was a discovery-based effort aimed at proteins expressed at a low level (*e.g*., TFs), we did not require proteins to be identified by multiple peptides to be included in downstream analysis.

We excluded nonquantified proteins from downstream analysis rather than impute missing values, resulting in a list of 5074 proteins. We first normalized the grouped abundance of each protein by the median value within a TMT channel (115) and then averaged biological replicates (Fig. 2*C*). Pairwise comparisons of global protein quantification across different conditions revealed the NoG-measurements poorly correlated with all conditions in which labeling occurred, as expected (Fig. 2*D*). In contrast, there was a high degree of correlation between the NoG + conditions and the pooled on target conditions (Fig. 2*E*). We considered all proteins that were enriched by at least 1.2-fold in NoG- *versus* the pooled labeling conditions and an adjusted *p*-value<0.05 to be contaminants and removed them from downstream analysis, resulting in 4377 proteins. We chose the FC threshold based on observations that isobaric tags can measure 1.2FC at the 10s-100s of femtomolar level in MS2-based quantification (116) compared with nM TF concentrations in eukaryotic cells. Our RTS-SPS-MS3 data acquisition is expected to have higher accuracy and precision (117). We used Bonferroni correction in generating the contaminant list in order to minimize the number of false positives identified as contaminants while correcting for multiple hypothesis testing (99).

### FOXP2 Promoter Proximal Proteome Is Enriched for TFs and Splicing Machinery

**Fig. 3:**
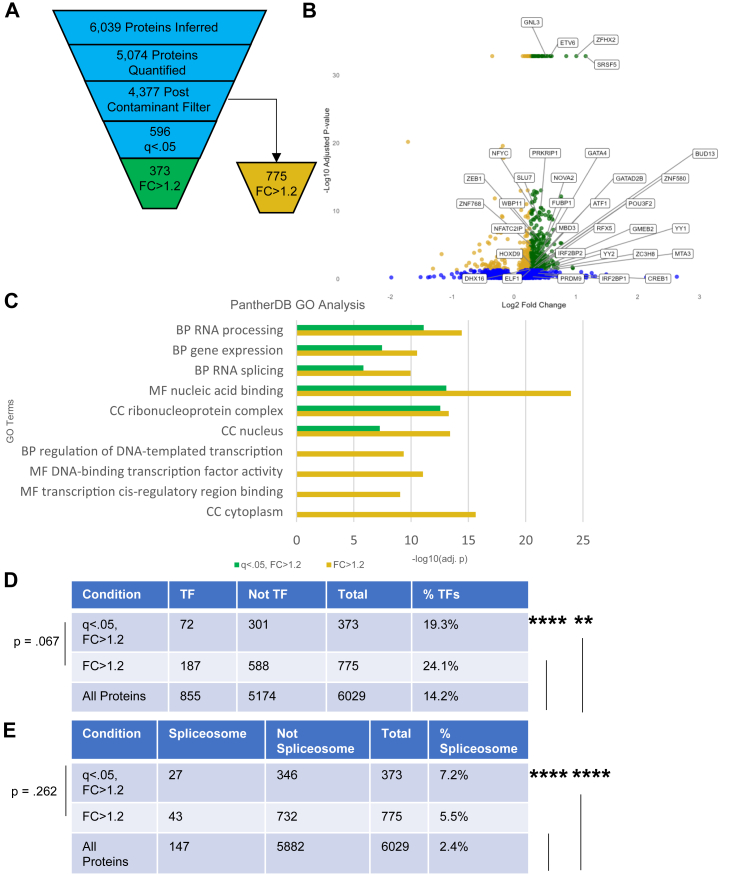
***FOXP2* promoter proximal proteome is enriched for transcription factors and splicing machinery.***A*, number of proteins remaining after each data analysis filter (quantification, NoG- contaminant list, *q*-value filter, and FC > 1.2). The number of proteins with FC > 1.2 post contaminant filter ignoring the *q*-value filter is also shown. *B*, volcano plot showing Log_2_FC(pooled on target *versus* NoG+) on the x-axis and -log_10_(*q*-value) on the y-axis. Proteins enriched with FC > 1.2 and q < 0.05 are highlighted in *green*, while proteins with *q* < 0.05 and FC < 1.2 are *golden*, and proteins with *q* > 0.05 are *blue*. Proteins presented in Table 1 are labeled. *C*, Gene Ontology enrichment analysis using PantherdbGO for proteins enriched by FC > 1.2 with (*green*) and without (*golden*) the *q*-value filter. *D*, contingency table for transcription factors identified in proteins enriched by FC > 1.2 with and without the *q*-value filter compared with all identified proteins. ∗∗*p* < 0.01 ∗∗∗∗*p* < 0.00001*. E,* contingency table for components of the spliceosome identified in proteins enriched by FC > 1.2 with and without the *q*-value filter compared with all identified proteins. ∗∗∗∗*p* < 0.00001. BP, biological process; CC, cellular component; FC, fold change; MF, molecular function; TF, transcription factor.

To determine the proteins enriched at the active *FOXP2* promoter, we compared the quantification between the pooled on-target conditions with the untargeted negative control (NoG+) using the Storey method to correct for multiple hypothesis testing (100, 101), generating 596 proteins with *q* < 0.05. Of these proteins, 373 were enriched with FC > 1.2. A recent report utilizing dCas9-APEX2 to identify a promoter-proximal proteome identified biologically relevant proteins that were nonsignificantly enriched (40). Ignoring significance, 775 proteins were enriched with FC > 1.2. After the filtration steps (Fig. 3*A*), we created a volcano plot to visualize the results (Fig. 3*B*).

We analyzed both sets of enriched proteins (FC > 1.2 with or without *q*-value filter) *via* gene ontology analysis using multiple tools (118, 119, 120, 121). Representative results from PantherdbGO are shown in Figure 3*C*, and results from all tools with -log_10_(adjusted *p*) > 6 are presented in Supplemental Table S4. Expected terms such as nuclear localization and mRNA processing indicate nuclear enrichment was successful. RNA splicing and related terms like ribonucleoprotein complex were enriched as well, potentially indicative of cotranscriptional splicing regulation (122) or accumulation of dCas9-APEX2 in the nucleolus (42). There generally was greater statistical significance in terms identified in the list of proteins with FC > 1.2 without the *q*-value filter; this list also had greater enrichment of terms related to sequence-specific TF binding and activity of RNA polymerase II, but it also included likely false positive terms like cytoplasm. We conclude that analyzing proteins enriched below statistical significance can unveil true promoter proximal proteins, but that results must be interpreted with caution and validated by orthogonal approaches.

In order to determine if we were able to successfully enrich TFs at the *FOXP2* locus, we compared our enriched proteins to the set of all human TFs. We first combined two previously compiled lists of human TFs and removed duplicates, generating a set of 2562 proteins (Supplemental Table S5) (123, 124). We then generated a contingency table to determine if the fraction of TFs in the enriched lists were statistically different from the entire set of proteins inferred in our mass spectrometry experiment (Fig. 3*D*). The fraction of TFs for the enriched proteins with (*q* < 0.05, FC > 1.2; 19.3%) and without the *q*-value filter (FC > 1.2; 24.1%) was significantly different from the total list of proteins (14.2%) by the χ^2^ test (*p* < 0.01 and *p* < 0.00001, respectively). The percent of components of the spliceosome (125) in the enriched lists with (*q* < 0.05, FC > 1.2; 7.3%, *p* < 0.00001) and without the *q*-value filter (FC > 1.2; 5.5%, *p* < 0.00001) was also significantly different from the spliceosome components in the whole set of inferred proteins (2.4%) (Fig. 3*E*) (Supplemental Table S5).

Within the set of significantly enriched TFs, we detected POU3F2, which has been shown to drive reporter gene expression from an intronic *FOXP2* enhancer and has a binding site highly conserved across vertebrates upstream of TSS1 (126, 127). We conclude that genetically targeted proximity labeling is able to successfully detect promoter-proximal proteomes, including classic sequence-specific TFs.

### Orthogonal Confirmation of Identified Proteins

Selected proteins enriched by FC > 1.2 at TSS1 are indicated in Table 1. To validate our findings, we utilized orthogonal approaches to benchmark our candidate transcriptional regulators identified by mass spectrometry.

**Table 1:** Selected mass spectrometry-enriched proteins at *FOXP2* TSS1

Protein	Class	Notes
POU3F2	TF	a ˆ Conserved binding site upstream of TSS1 (126)
GNL3	TF	a ˆ Member of Wnt/β-catenin pathway (143)
NFYC	TF	a ˆ b
GMEB2	TF	a ˆ b
RFX5	TF	a ˆ b
HOXD9	TF	a ˆ b
ELF1	TF	a ˆ b
ETV6	TF	a ˆ
ZEB1	TF	a ˆ
GATA4	TF	a ˆ
FUBP1	TF	a ˆ
ZC3H8	TF	a ˆ
ATF1	TF	a ˆ b Co-binds with CREB1
CREB1	TF	ˆ b Co-binds with ATF1, binds to *FOXP2* e330 in ChIP-chip analysis (132)
YY1	TF	a ˆ b ENCODE ChIP-Seq peak at TSS1
YY2	TF	a ˆ ENCODE ChIP-Seq peak at TSS1
ZFHX2	TF	a ˆ ENCODE ChIP-Seq peak at TSS1
ZNF768	TF	a ˆ ENCODE ChIP-Seq peak at TSS1
ZNF580	TF	a ˆ ENCODE ChIP-Seq peak at TSS1
ZEB1	TF	a ˆ ENCODE ChIP-Seq peak at TSS1
NFATC2IP	TF	a ˆ NFATC1 predicted by TFBSPred; NFATC proteins (1 and 2) impact β-cell proliferation through FOXP-dependent mechanism (131)
IRF2BP2	TF	a ˆ Partner protein IRF2 predicted by PROMO
IRF2BP1	TF	ˆ Partner protein IRF2 predicted by PROMO
SRSF5	Spliceosome	a ˆ
PRKRIP1	Spliceosome	a ˆ
SLU7	Spliceosome	a ˆ
NOVA2	Spliceosome	a ˆ
WBP11	Spliceosome	a ˆ
BUD13	Spliceosome	a ˆ
DHX16	Spliceosome	a ˆ
PRDM9	Chromatin remodeler	ˆ Validated H3K4Me_2_ → H3K4Me_3_ activity, consistent with promoter chromatin state
GATAD2B	Chromatin remodeler	a ˆ NuRD complex member
MBD2	Chromatin remodeler	a ˆ NuRD complex member
MBD3	Chromatin remodeler	a ˆ NuRD complex member
MTA3	Chromatin remodeler	ˆ NuRD complex member

a *q* < .05, ˆ FC > 1.2.

b Predicted by computational TF tool.

### Computationally Predicted TF Binding to the FOXP2 Locus

We first used two separate computational tools that predict potential TF binding sites at DNA regions, TFBSPred (128) and PROMO (129, 130). TFBSPred predicted potential binding of the mass spectrometry-identified hits NFYAˆ, NFYC∗ˆ, KLF9ˆ, RFX5∗ˆ, ATF1∗ˆ, CREB1ˆ, GMEB2∗ˆ, VEZF1ˆ, CEBPA∗, and NR2F2∗ˆ (∗*q* < 0.05, ˆFC > 1.2). The protein NFATC1 was also predicted by TFBSPred. Both NFATC1 and NFATC2 have been shown to impact β-cell proliferation through a mechanism relying on FOXP proteins from a FOXP1/2/4 triple knockout model (131). Interestingly, although NFATC1 quantification was not significant and unchanged (*q* > 0.05, FC = 1.05) and we did not detect NFATC2, the related NFATC2IP∗ˆ was significantly enriched.

The mass spectrometry-identified hits NFIX∗, MAZˆ, NFYC∗ˆ, NFYAˆ, YY1∗ˆ, ATF1∗ˆ, CREB1ˆ, HOXD9∗ˆ, and ELF1∗ˆ were identified as potential binders by the PROMO web tool. Our mass spectrometry results detected the PROMO-predicted protein IRF2, but it was not enriched. However, related proteins IRF2BP1ˆ and IRF2BP2∗ˆ were both enriched *via* quantitative mass spectrometry (1.40FC and 1.30FC, respectively). The output of predicted binders from both tools is included as Supplemental Table S6. We note that the tools considered all possible human TFs across all cell types, including factors not expressed in HEK293 cells. Therefore, we would not expect to have high coverage of the predicted binders.

The known coregulators ATF1 and CREB1 were identified by both computational tools and our mass spectrometry results. Consistent with these observations, a ChIP-chip analysis of CREB1 in HEK cells showed binding at the *FOXP2* locus (132). Notably, the sequence in the microarray analysis was included before identification of the active upstream promoter targeted in our experiments. It instead represents a downstream region comprising two alternative promoters and a conserved enhancer that has been demonstrated to form an active chromatin loop with TSS1 in HEK293 cells (86) (*vide infra*).

### ENCODE ChIP-Seq Database

**Fig. 4:**
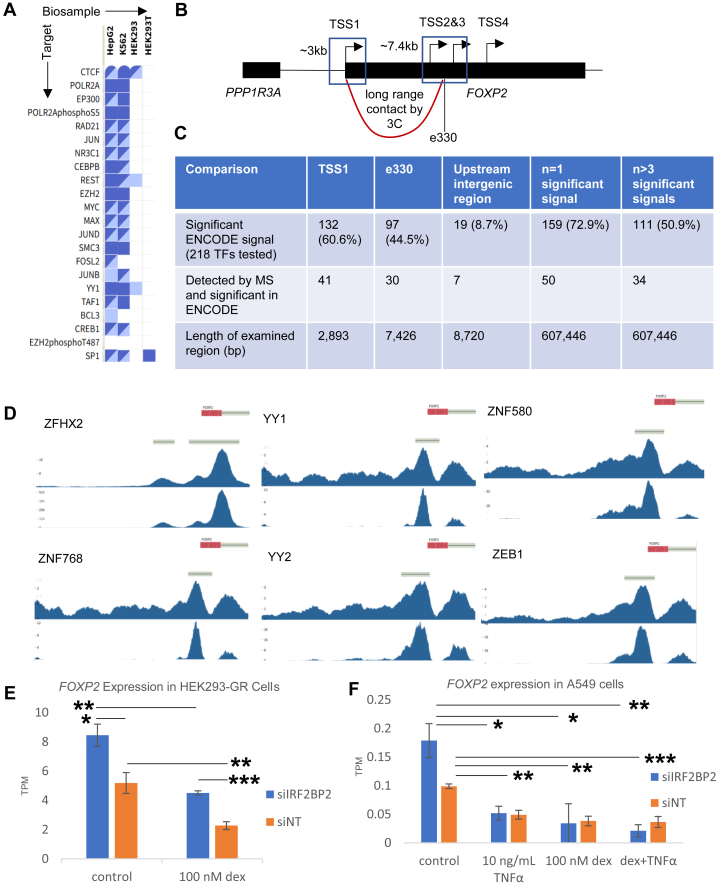
**Orthogonal confirmation of identified proteins.***A*, ENCODE ChIP-Seq Data Matrix available at encodeproject.org. Filtered to show transcription factors in human cell lines HepG2, K562, HEK293, and HEK293T. *B*, gene body diagram showing *FOXP2* genomic regions examined in ENCODE ChIP-Seq database. TSS1 = hg38 chr7: 114084641-114087534; e330 = hg38 chr7:114411165-114418591, comprising TSS2&3 and a conserved enhancer. A 3C study has indicated these regions form a long range chromatin loop (86), indicated by a *red line*. *C,* number of transcription factors bound at each indicated region of *FOXP2* from ENCODE ChIP-Seq database and corresponding mass spectrometry results. See also Supplemental Table S7 for detailed comparison of ENCODE results to mass spectrometry results. *D*, ENCODE ChIP-Seq tracks of the six proteins with called peaks that were enriched in the mass spectrometry dataset (*q* < 0.05, FC > 1.2). Each track shows the *FOXP2* gene body, the irreproducible discovery rate called peak thresholds, the fold change of the indicated transcription factor over control, and the associated *p*-value. Each transcription factor has its own data-dependent scale bar for fold change and *p*-value. *E,* expression of *FOXP2* in transcripts per million (TPM) in HEK293 cells constitutively expressing the glucocorticoid receptor (GR). Expression of *FOXP2* transcripts is increased upon siRNA knockdown of IRF2BP2 and decreased upon activation of GR signaling by dexamethasone. Error bars = SEM, n = 4. ∗*p* < 0.05, ∗∗*p* < 0.01, ∗∗∗*p* < 0.001*. F,* expression of *FOXP2* in TPM in A549 cells with siRNA knockdown of IRF2BP2 or nontargeting (NT) with or without activation of GR and/or TNFα signaling. Error bars = SEM, n = 3. ∗*p* < 0.05, ∗∗*p* < 0.01, ∗∗∗*p* < 0.001. 3C, chromatin conformation capture; ChIP, chromatin immunoprecipitation; ENCODE, Encyclopedia of DNA Elements; TF, transcription factor; TSS, transcription start site.

We sought to further validate our mass spectrometry results by identifying true positive proteins that bind to the *FOXP2* promoter in HEK293 cells. The gold standard for identifying protein–DNA interactions in cells is ChIP-Seq and related approaches. The ENCODE database has a collection of ChIP-Seq experiments targeting a diversity of TFs across many cell lines (Fig. 4*A*). We analyzed the 223 ChIP-Seq experiments performed in HEK293(T) cells to determine TF and histone post-translational modification status at the *FOXP2* gene (Fig. 4B). We analyzed binding of factors at the 2893 bp region of TSS1 that has been validated as an active promoter by PolII binding and luciferase assays. A chromatin conformation capture (3C) study (86) has demonstrated long range contact between TSS1 and the 7426 bp region that comprises TSS2, TSS3, and a highly conserved enhancer 330 kbp away from TSS1, which contains the sequence included in the microarray analysis referenced above; we therefore included 7.4 kbp regulatory region (hereafter: e330) in our analysis. We also considered the intergenic region upstream of TSS1 between *FOXP2* and *PPP1R3A*, which includes a purported human-specific enhancer upstream of TSS1, and whether there were more than three significant peaks in the *FOXP2* gene body (n > 3) to more comprehensively characterize chromatin interactors at the genetic locus in HEK293 cells. We compared the presence of significant ChIP-Seq signals at these loci in the ENCODE database with our mass spectrometry data in Supplemental Table S7.

Our targeted promoter showed H3K4Me_3_ and H3K27Ac histone marks, consistent with an active TSS (133). These histone modifications were present at e330 in addition to H3K4Me_1_, in line with an active enhancer region. Consistent with the observed ChIP-Seq-based chromatin state of our targeted promoter, we detected by MS the nonsignificantly enriched chromatin remodeler PRDM9ˆ, which has validated H3K4Me_2_ → H3K4Me_3_ methyltransferase activity (134). We also detected members of the nucleosome remodeling and histone deacetylation (NuRD) complex GATAD2B∗ˆ, MBD2∗ˆ, MBD3∗ˆ, and MTA3ˆ (135). The NuRD complex has been demonstrated to be associated with H3K4Me_3_ histone modifications at poised bivalent sites and to bind at the promoter of active genes (136, 137). By contrast, we failed to detect enrichment of multiple members of the SWI/SNF, ISWI, and INO80 families of chromatin remodelers, suggesting the NuRD complex may regulate the epigenetic state of the *FOXP2* promoter in HEK293 cells.

Of the 218 TFs studied in HEK cells by ENCODE, 132 (60.6%) showed significant ChIP-Seq signals at TSS1 (Fig. 4*C*). At e330, 97 (44.5%) TFs showed a significant signal. Only 19 (8.7%) showed binding in the upstream intergenic region between TSS1 and *PPP1R3A*. There were 159 factors (72.9%) with significant signals in the *FOXP2* gene body, and only 111 (50.9%) had more than three significant signals. Most of the TFs detected at e330 overlapped with those found at TSS1, with the exception of 12 proteins that had no promoter peak but were bound to the enhancer (FOXA1, TRIM28, ZNF384, MEIS1, ZFP3, ZNF34, ZNF362, ZNF624, AEBP2, GFI1B, GLI2, and ZNF654). Of the 218 TFs that have ChIP-Seq experiments in HEK293 cells in the ENCODE database, we detected 72 by mass spectrometry. We detected 41 of the TFs with ChIP-Seq signals at TSS1 in our mass spectrometry dataset, suggesting that 91 ChIP-Seq positive TFs in the ENCODE database (68.9%) were undetected as false negatives. Fifty-seven percent of the mass spectrometry detected TFs with ENCODE data (41/72) were true positive binders at TSS1 before performing bioinformatic analysis on the dataset.

Of the 72 ENCODE TFs identified in our data set of 6029 detected proteins, only 58 were enriched in labeled conditions *versus* NoG-; the majority of proteins eliminated (12/14, 86%) were not quantified at all, indicating they were present near the detection limit of the instrument rather than being enriched in NoG-. There were 13 TFs with *q* < 0.05, of which 11 had FC > 1.2. Only eight of those 13 (61.5%) had ChIP-Seq signals, and the two eliminated by the FC > 1.2 filter (TARDBP and PKNOX1) were true positives in ENCODE ChIP-Seq. The ChIP-Seq tracks of the six proteins with *q* < 0.05, FC > 1.2 and significant ENCODE ChIP-Seq signals are shown in Figure 4*D*. Comparing ENCODE results to the list of all proteins with FC > 1.2 without the *q*-filter, 25 of 57 were enriched, but only 14 showed signals at TSS1 (56%). Of the 11 false positives, there were three TFs with peaks at e330 (TRIM28, ZNF384, and MEIS1).

### Comparison to Known Regulators of FOXP2 Expression

We further compared our mass spectrometry data and results from the ENCODE database with the few known regulators of *FOXP2* expression. One of the most well-known and best characterized regulators of *FOXP2* expression is the Wnt/β-catenin TF LEF1. Experiments in zebrafish embryos have demonstrated that lef1 directly binds to and regulates the expression of *foxp2* (138). In contrast, LEF1 was detected and passed the NoG-contaminant filter, indicating nuclear expression, but it was not enriched in our targeted mass spectrometry results. Furthermore, ENCODE showed no significant ChIP-Seq signal at TSS1 or e330 in HEK293 cells. This result highlights the need for care in transferring observations of transcriptional regulation across cell types. On the other hand, the TCF/LEF TF family member TCF7L2 showed a significant ChIP-Seq signal at TSS1 and e330. Although we detected TCF7L2 in our inferred proteins, it was not significant and was more enriched in NoG + conditions compared to on target. However, analysis of the PSMs showed that the only identified peptide was shared with LEF1. This observation highlights the difficulty in the protein inference problem for which there are multiple approaches and no commonly accepted best practice (139, 140, 141, 142). It is noteworthy that one of the top hits in our list (top 10 FC *versus* NoG + for *q* < 0.05), GNL3, is a component of the Wnt/β-catenin signaling pathway (143).

The TF FOXK2 was predicted to bind to the *FOXP2* promoter by TFBSPred and showed a significant signal in the ENCODE ChIP-Seq database at TSS1. However, there was no difference between on and off-target conditions in our quantitative dataset (FC = 1.02). Furthermore, the protein ZBTB20 showed a significant ChIP-Seq signal, and the mouse homolog Zbtb20 has been shown to bind to and control *FoxP2* expression in the developing mouse brain (144), but we failed to detect it in our mass spectrometry dataset. The homolog of the human protein PAX6 has been identified as binding to the *foxp2* locus and impacting gene expression in zebrafish models (145). We detected this protein, but it was not enriched in on target conditions (FC = 1.05), although it was not tested in HEK293 cells by ENCODE. Each of these observations highlights that a lack of detection or enrichment in our mass spectrometry dataset does not preclude a protein from being a member of the *FOXP2* promoter-proximal proteome.

### IRF2BP2 and Glucocorticoid Signaling Are Negative Regulators of FOXP2 Transcription

Given that there can be limited overlap between TF binding and regulatory activity (146), we sought to demonstrate a direct impact on transcriptional output rather than simply a binding interaction at the *FOXP2* promoter from within our proposed candidate regulators. The protein IRF2BP2 was significantly enriched in our proteomic assay and has been identified as an IRF2-dependent transcriptional repressor (147, 148). Given that IRF2BP1 was also enriched (nonsignificantly) and IRF2 was predicted from the PROMO binding tool, we sought to characterize effects of IRF2BP2 on *FOXP2* expression. Characterization of IRF2BP2 genome-wide binding and effects of its depletion on transcription have been reported previously in HEK cells that constitutively express the glucocorticoid receptor (149).

As shown in Figure 4*E*, siRNA knockdown of IRF2BP2 leads to a significant 1.6-fold increase in expression of *FOXP2* compared with a nontargeting control (siNT). This effect persisted when glucocorticoid signaling was activated by addition of 100 nM dexamethasone. Notably, activation of glucocorticoid signaling by dexamethasone significantly decreased *FOXP2* expression in both siIRF2BP2 and siNT conditions. There were three identified peaks in the *FOXP2* gene body for IRF2BP2 binding *via* ChIP-Seq in control conditions and only one peak with dexamethasone treatment, although the control peaks sat between TSS1 and e330 and did not include our targeted promoter. Conspicuously, one of the three peaks in control conditions resides approximately 56 kbp downstream of the targeted promoter, a region that forms a weak chromatin loop (*i.e.,* close spatial relationship) with TSS1 across neuronal cell types in 3C experiments (86). The peak in dexamethasone-treated cells was more than 400 kbp downstream of TSS1 and did not have evidence for long range chromatin looping in any cell types since it was outside the interrogated enhancer-promoter pairs in the 3C study.

To determine whether these observations were translatable to other cellular contexts, we compared *FOXP2* expression before and after knockdown of IRF2BP2 in the lung cancer cell line A549 from the same study (Fig. 4*F*). FOXP2 is only weakly expressed at the transcript and protein levels in A549 cells, lowering power to call statistical significance (note scale bar differences between 4E and 4F). This downregulation is tied to increased aggressiveness of lung cancer (150). Knockdown of IRF2BP2 led to a 1.8-fold increase in *FOXP2* expression that approached but did not reach significance at the *p* < 0.05 level compared to siNT conditions (*p* = 0.0558). Treatment with dexamethasone, TNFα, or a combination of both significantly decreased *FOXP2* expression. These changes did not differ between siIRF2BP2 and siNT conditions (*p* > 0.3), and there were no detectable differences among the individual or combined treatments. We conclude that IRF2BP2 and glucocorticoid signaling can negatively regulate *FOXP2* expression across cell types.

### Assessment of Proteomics Locus Specificity by Off Target Locus ENCODE Comparison

To ascertain the locus specificity of our proteomics data, we repeated our ENCODE analysis at several off target regions and compared the results to our MS-detected proteins. Since long-range DNA contacts are typically intrachromosomal, we chose genomic loci outside chromosome 7 that, to the best of our knowledge, have no implications for co-regulation with *FOXP2* based on the literature to date. Each analyzed locus was approximately 3 kbp in length and comprised the furthest upstream promoter of a gene to align with the analysis of *FOXP2* TSS1.

**Fig. 5:**
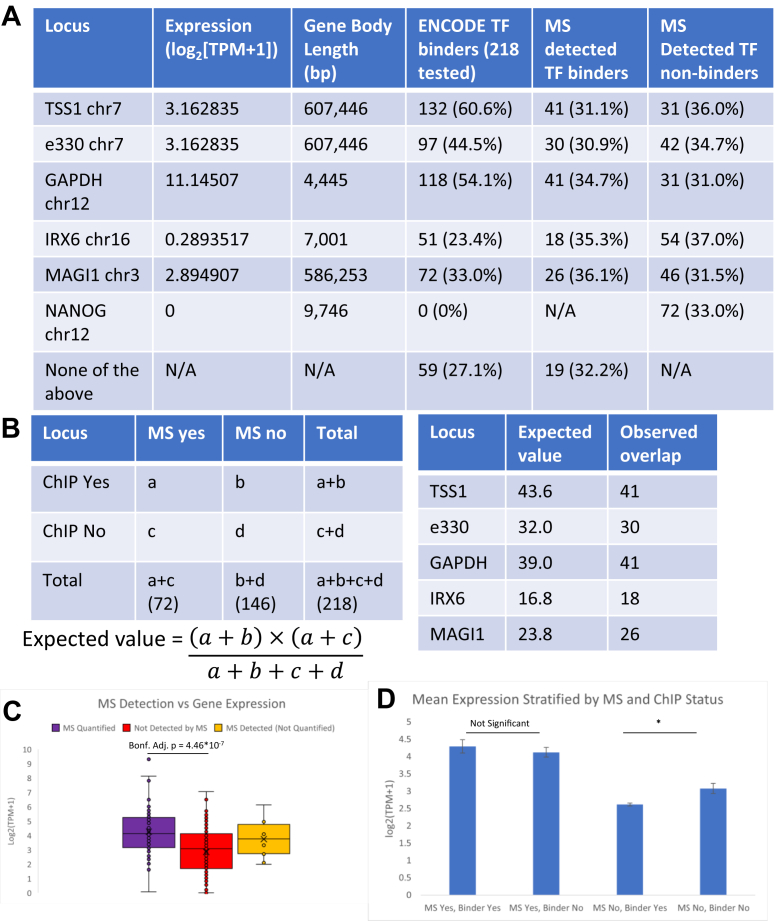
**Assessment of proteomics locus specificity by off target locus ENCODE comparison.***A*, expression, ENCODE TF binding status in HEK293 cells, and MS TF detection status for FOXP2 TSS1 and e330 and promoters of GAPDH, IRX6, MAGI1, and NANOG. *B*, formula for calculating expected value for overlap of ChIP- and MS-positive TFs with expected *versus* observed values at each locus. *C*, mean gene expression for proteins that MS detected and quantified (n = 60), did not detect (n = 146), or detected but did not quantify (n = 12). Quantified *versus* undetected Bonf. Adj. *p* = 4.46∗10^-7^. *D*, average expression levels measured in log_2_(TPM+1) pooled across loci stratified by MS detection and ChIP-Seq binding status. ∗Bonf. Adj. *p* < 0.05.

We chose genes to represent a variety of transcriptional states. Accordingly, the transcript per million (TPM)-based expression levels in HEK293 cells (log_2_[TPM+1] according to DepMap (151)) ranged from silent heterochromatin in *NANOG* (0) to lowly transcribed *IRX6* (0.2893517) to moderately transcribed *MAGI1* (2.894907) to the housekeeping *GAPDH* (11.14507) as compared to the moderately transcribed *FOXP2* (3.162835). In comparison to the 132 ENCODE TFs binding to *FOXP2* TSS1 (60.6%) and 97 TFs binding e330 (44.5%), 0 TFs bind *NANOG* (0%), 51 bind *IRX6* (23.4%), 72 bind *MAGI1* (33.0%), and 118 bind *GAPDH* (54.1%) (Fig. 5*A*).

We first performed a locus specificity analysis based on the ENCODE ChIP-Seq data. Global analysis of binary binding data at each locus showed a significant difference in TF binding proportion for at least one locus, even when removing the no-binder *NANOG* condition (Cochran’s Q(df), Q(5) = 306.9, *p* = 3.215∗10^−64^; Q(4) = 129.1, *p* = 6.207∗10^−27^ removing *NANOG*) (supplemental Table S8). We found that the proportion of TF binders at each locus in the ENCODE ChIP-Seq data showed significant difference for all pairwise locus comparisons except for TSS1-*GAPDH* (Bonf. Adj. *p* = 0.921), e330-*GAPDH* (Bonf. Adj. *p* = 0.122), and e330-*MAGI1* (Bonf. Adj. *p* = 0.0584) in follow-up McNemar’s tests (Supplemental Table S8).

We created contingency tables for each locus to determine if the 72 TFs detected by MS with ENCODE ChIP-Seq data showed enrichment for binders in either on target or off target conditions (Supplemental Table S8). The expected overlap between MS detection and ChIP-Seq positive TFs did not differ significantly at any analyzed locus (Fig. 5*B*) (Fisher’s Exact test, *p* > 0.4 for all loci). We detected approximately a third of TFs with ENCODE data whether looking at locus specific binders (range 30.9–36.1%), locus-specific nonbinders (range 31.0–37.0%), or TFs that did not bind any of the analyzed loci (32.2%) (Fig. 5*A*).

We hypothesized that the similar proportion of detection across all analyzed loci reflected both inherent MS sensitivity limits and underlying biology. We compared the expression levels for ENCODE TFs based on DepMap log_2_[TPM+1] in HEK293 cells that our MS data acquisition did not detect (n = 146), detected but did not quantify (n = 12), or detected and quantified (n = 60) (Fig. 5*C*). Proteins we did not detect by MS showed significantly lower expression (mean = 2.861387, SD = 1.6202) compared to those we detected and quantified (mean = 4.282906, SD = 1.644012) (Bonf. Adj. *p* = 4.46∗10^−7^). There was not a significant difference in average expression for TFs that were detected but not quantified (mean = 3.748952, SD = 1.194371) *versus* TFs that were either quantified (Bonf. Adj. *p* = 0.652) or not detected (Bonf. Adj. *p* = 0.109). For the 13 lowest expression TFs we quantified by MS (log_2_[TPM+1] < 3), TSS1 showed the highest proportion of binders (9, 69.2%), followed by e330 and *GAPDH* (8, 61.5%), then *MAGI1* (6, 46.2%) and *IRX6* (4, 30.8%).

To assess locus specificity of our proteomics data in light of expression-based detection limitations, we compared averaged log_2_(TPM+1) expression data for TFs at each locus stratified by MS-detection status and ChIP-Seq binding status, excluding *NANOG* due to a lack of binders (Fig. 5*D*) (Supplemental Table S8). Average expression across all loci for the four conditions significantly differed for all pairwise comparisons with a difference in MS detection status, as expected. TFs we detected by MS did not show a significant difference in expression based on ChIP-Seq binding status. On the other hand, TFs we did not detect by MS showed significantly lower expression comparing binders and nonbinders (Bonf. Adj. *p* < 0.05). In a locus-specific analysis, mean expression for TFs not detected by MS showed lower expression for binders compared to nonbinders only at the targeted TSS1 locus (Bonf. Adj. *p* < 0.05), while expression for other loci did not differ significantly with binding status (Bonf. Adj. *p* > 0.1).

## Discussion

The presence of multiple promoters within a gene permits regulatory systems that can incorporate complex logic operations for precise operation of single copy gene dosage (152). While synthetic biologists are just beginning to harness these principles for designed cellular phenotypes, evolution has continuously optimized gene circuits as long as they have existed (153). These tightly regulated genetic circuits can lead to discordance between mRNA and protein levels, emphasizing the need for proteomic measurements to accurately capture cell states (12, 13, 14). The *FOXP2* locus is an outlier within the genome, being enriched with more than 10 ultraconserved noncoding regulatory regions conserved across vertebrates but not invertebrates (75) and harboring multiple alternative promoters active across different conditions (85). This high conservation with multiple promoters allows FOXP2 to fill many roles, from neuronal expression related to vocal learning (74) to body plan development and jaw formation (82, 154).

We used genetically targeted proximity labeling with dCas9-APEX2 to covalently tag with biotin the promoter-proximal proteome of the single copy locus *FOXP2* at the upstream regulatory region active in that and many other cell types (TSS1) (85). The extensive washing to remove background contaminants coupled with quantitative mass spectrometry *via* online 2D-LC with TMT-RTS-SPS-MS3 enabled by a tribrid mass spectrometer (Fig. 2*A*) allowed us to attain deep proteome coverage and identify 373 proteins significantly enriched at the *FOXP2* promoter (Storey-*q* <0.05, FC > 1.2), including 72 TFs. Many of these candidate transcriptional regulators have orthogonal evidence of biological relevance—from previous literature evidence to computationally predicted TF binding to experimental ENCODE ChIP-Seq data. A great deal of effort has gone into generating mice with disruptions to FoxP2 expression in a bid to understand its biological function (155, 156, 157, 158). The candidate regulators identified in this study can be further used for hypothesis generation to uncover the upstream mediators of FoxP2 effects in different conditions and developmental time points.

All identified proteins are only candidate regulators until confirmed through orthogonal lines of evidence. We leveraged the ENCODE database to provide direct evidence for or against factors that were present in both datasets, giving an important benchmark for our proteomic results and bioinformatic analysis pipeline. Although there were more than 200 TF ChIP-Seq experiments in HEK293 cells, some of our candidate regulators we detected and enriched by MS that were not tested in HEK cells had been tested in other cell lines. Future locus-specific chromatin isolation studies would benefit from utilizing one of the ENCODE ‘depth’ cell lines, like HepG2 or K562 (Fig. 4*A*), if they accurately recapitulate the transcriptional conditions of interest to researchers.

Confirmation of candidate regulators as binders *via* ChIP-Seq or ChIP-qPCR is a resource and labor-intensive process that also requires the existence of ChIP-grade antibodies. Contemporary locus-specific chromatin studies often follow up on only a few biologically informed hits and deeply characterize them (28, 39, 40, 44). A fruitful frontier is determining changes in proteome composition at a targeted locus in response to experimental perturbations (41, 42, 43, 159). Mass spectrometry provides rich, multidimensional data of measured peptides and their quantification to the protein level which can be used to prioritize hits for follow up in a discovery-based manner. Some of the false positives identified in our mass spectrometry dataset that did not show signals in the ENCODE database had weak underlying PSM data (*e.g*., identified by a single shared peptide), highlighting the utility of incorporating the mass spectrometry data at all levels for decision making. There is much to be gained from systematic analysis of proteomics datasets in addition to targeted follow ups. Caution must be taken, however, especially with results from labs without deep MS-based proteomics experience—it is not uncommon in the proteomics field to observe uncorrected *p* values at the protein quantification level (101) despite stringent, data-driven FDR at the PSM, peptide, and protein identification levels. With calls from active practitioners (54, 99, 160) and options for multiple hypothesis correction in most MS data analysis tools, the proteomics community is beginning to adopt statistical strategies for multiple hypothesis testing in protein bioinformatic analysis, but it is by no means universal.

It is noteworthy that LEF1 showed no signal at TSS1 or e330 in ChIP-Seq given its demonstrated role in regulating *foxp2* expression in developing zebrafish (138). This was not due to lack of expression—we detected LEF1, and it was enriched relative to NoG-conditions. Nuclear expression of a TF coupled to open chromatin at an actively transcribed gene that it is known to regulate under certain conditions is not sufficient to induce binding. It is possible that LEF1 can be recruited to the *FOXP2* locus in HEK293 cells under the appropriate signaling conditions. Alternatively, LEF1 may need coregulators to bind to *FOXP2* that are not expressed in HEK293 cells. Another possibility is that LEF1 binding and regulation of *FOXP2* occurs at genomic elements other than the upstream transcribed promoter or active enhancer 330 kb downstream or that LEF1 regulates *FOXP2* expression without a direct binding interaction (146). Other components of the Wnt/β-catenin signaling pathway do have evidence for interaction with *FOXP2* TSS1 in HEK cells—TCF7L2 binding at TSS1 was significant according to ENCODE ChIP-Seq, and GNL3 was one of our top significantly enriched hits.

We identified two novel negative regulators of *FOXP2*, IRF2BP2 and glucocorticoid signaling. Knockdown of IRF2BP2 with siRNA increases expression of *FOXP2* at the transcript level, while activation of glucocorticoid signaling by dexamethasone decreases it. These effects appear additive, with the lowest expression levels observed in conditions with expression of IRF2BP2 and activation of glucocorticoid signaling. In addition to IRF2BP2 being significantly enriched in our proteomics results, the partner proteins IRF2BP1 and IRF2 were nonsignificantly enriched and predicted from TF binding tools, respectively. When choosing hits for labor-intensive follow-up validation, it is prudent to pursue proteins that are part of complexes with multiple identified members. The known coregulators CREB and ATF1, both enriched and with computational evidence, have literature evidence for binding to *FOXP2* (132). Multiple members of the NuRD complex were enriched, while other families of chromatin remodeling complexes were not identified. Leveraging prior knowledge on association in shared protein complexes can improve the likelihood of biological relevance.

We found that expression levels for ENCODE TFs significantly correlated with MS-detection status, with more lowly expressed genes less likely to be detected. There was no difference in mean expression based on ChIP-Seq binding status for proteins detected by MS. For ENCODE proteins we did not detect by MS, the mean expression of binders was significantly lower than for nonbinders. Stratifying by locus, only the on target TSS1 showed lower expression for binders, while the expression levels for TF binders and nonbinders did not differ significantly at any of the other analyzed loci. These results indicate that genetically targeted proximity labeling is specific for a targeted region, but sensitivity is still limited. Our off target ENCODE analysis also suggests that highly transcribed genes are not ideal controls for locus specificity analysis given that ChIP-Seq data showed no significant difference in proportion of TF binders between TSS1-*GAPDH* and e330-*GAPDH*. This observation is in line with results from the 4D Nucleome project, which indicate highly expressed housekeeping genes form multiple long-range enhancer–promoter interactions (161). It is interesting to note that *FOXP2* has the highest number of ENCODE TF binders at analyzed loci, exceeding the housekeeping *GAPDH* and giving further evidence for its complex layers of regulation.

### Limitations of the Study

One limitation of this study is the use of an untargeted dCas9 as a negative control rather than a nontargeting gRNA containing a sequence not found in the human genome. The dynamics of dCas9 interrogating the genome differs in the presence and absence of a gRNA (162). An off-target locus (36, 159) or an alternative locus of interest for comparative studies (41) can also be used as a control to account for the high background in proximity labeling studies. Despite the high background, proximity labeling is a valuable approach that provides complementary information to affinity purification, including the ability to detect lower abundance proteins (163). A snapshot of chromatin regulators within an approximately 400 bp radius (38) over the course of a minute helps unravel the dynamic processes underlying gene regulation, including the ability to detect proteins that may not be cross-linked by formaldehyde or that associate with the targeted locus transiently during the labeling period.

Our on-bead digestion protocol leaves behind the residues and associated proteolytic fragments that were biotinylated in our sample preparation (164) (canonically tyrosine, but cysteine and lysine labeling have been observed as well with peroxidase-based methods (165)), compounding the protein inference problem by removing potentially informative peptides from what we injected on the mass spectrometer (166, 167). Using desthiobiotin (26, 168) or a clickable biotin analog with a cleavable linker to release labeled proteins from the streptavidin beads before digestion could capture those peptides while preventing the unacceptable sample loss for low-input studies that occurs with repeated manipulations on the microscale. Such an approach could be adapted in principle to top-down proteomics protocols to study alternative proteoforms interacting with a promoter (169).

Another shortcoming is that this is necessarily a bulk measurement averaged across many cells. It is unlikely that all the identified regulators co-occupy the *FOXP2* promoter at the same time in the same cell, obscuring the important biological insights that can be obtained at the single cell level (14, 170). Understanding which factors cobind or which are mutually exclusive to each other is not possible with this dataset alone. Split enzymes or FRET assays could be an approach to test cobinding of specific factors.

Another issue is that cells were not synchronized. The TF YY1, significantly enriched in our dataset, predicted by PROMO, and with a significant ChIP-Seq signal at TSS1 according to ENCODE (Fig. 4*D*), has been shown to have differing effects on its role in establishing and maintaining chromatin loops based on the cell cycle at some genomic locations (171). The interaction of FOXP2 itself with DNA is also tightly regulated and controlled throughout the cell cycle (172). Synchronizing cells could help ensure promoters of interest have similar chromatin states.

There were many false negatives (nearly 70% of ENCODE-positive TFs) despite the high initial input of cells (∼6∗10^8^ on-target chromatin equivalents). Some TFs we detected were not quantified, indicating they were present below the limit of quantification, and detection was significantly correlated with TF expression level with a uniform detection rate of approximately one-third (Fig. 5*C*). Utilizing mass spectrometry approaches like parallel reaction monitoring could help identify candidate regulators at an isolated genomic locus in a hypothesis-driven, targeted approach (173).

It is well appreciated that TFs can undergo extensive posttranslational modifications that impact subcellular localization, interaction partners, and regulatory function (132, 133, 172, 174, 175). We used a closed search strategy and did not specify functionally relevant posttranslational modifications like phosphorylation, methylation, or lysine acetylation in analyzing our data. An open search, spectrum-centric strategy can identify and localize posttranslational modifications even if they have not previously been identified (176, 177, 178). Such an approach could in principle identify peptides and proteins within this dataset containing posttranslational modifications related to transcriptional regulatory function, potentially including histone modifications (179).

Generating enough starting material for deep proteome coverage is labor intensive. Contemporary locus-specific proteomics experiments often target repetitive regions like telomeres (92 copies of repetitive sequence per cell) (26, 27, 34, 36, 37, 42), centromeres (46 copies of repetitive sequence per cell) (34, 37, 42, 180, 181), or LINE-1 transcribed promoters (80–100 copies per cell) (181, 182). Another approach is to use reporter plasmids present at a higher copy number to increase intrinsic signal (40, 183) or to use a model system with a smaller genome to reduce background (184), examples of single-copy locus enrichment in human systems (two copies per cell) notwithstanding (28, 29, 38, 39, 42, 43, 44, 159). Strategies to reduce necessary input volume are needed to enable more widespread adoption of promoter-pulldown proteomics. Single cell proteomics approaches have utilized a TMT-based signal boosting approach to generate enough material for analysis (185). Adaptation of that paradigm for promoter-proximal proteomes is a promising avenue for development. The data-dependent acquisition approach used in this study samples only a fraction of the ions that elute from the LC for MS analysis stochastically. Data-independent acquisition approaches (159, 181), especially when coupled with parallel accumulation-serial fragmentation enabled by ion mobility separation (186, 187), can sample all ions eluting off the LC and provide deep proteome coverage on material-limited samples, giving another potential avenue to reduce sample input requirements. For promoter occupancy studies on very highly conserved genes, Epi-Decoder provides an interesting proteomics-by-sequencing approach that can be used in yeast (188).

## Conclusion

The majority of disease-risk variants identified in genome-wide association studies map to noncoding regions (189). Understanding how these genetic variants give rise to different phenotypes and impact human health requires being able to determine chromatin-associated proteins and how they differ across sequence diversity (190). Forward genetic studies relying on targeted antibodies to determine genome-wide association of disease relevant factors have been leveraged to great effect. Even with increasingly sophisticated protocols that allow individual labs to collect data at a scale previously accessible only to multi-institution consortia (191), these forward genetic studies are intrinsically limited by the availability of high-quality antibodies. Genetically targeted proximity labeling coupled to quantitative mass spectrometry proteomics is a powerful approach for discovery-based reverse genetics to determine the phenotypic impact of sequence variation without the need for ChIP-grade antibodies or a prior hypothesis of molecular mediators. The biochemical and analytical challenges can also serve as a Muse for development of new and improved proteomics instrumentation, sample preparation, data acquisition, and analysis protocols (192, 193).

## Data availability

Mass spectrometry data described in this communication have been deposited to the MassIVE repository partner of the ProteomeXchange Consortium available at massive.ucsd.edu with the identifier MSV000098217. Experiment identifiers and contributing labs for the 223 ENCODE ChIP-Seq experiments utilized in this study are enumerated in Table S7. Data for IRF2BP2 knockdown and ChIP-Seq were obtained through the NCBI GEO with accession identifier GSE124636.

## Supplemental data

This article contains supplemental data.

## Conflict of interest

M. P. S. is a co-founder and scientific advisor for Crosshair Therapeutics, Exposomics, Filtricine, Fodsel, Iollo, InVu Health, January AI, Marble Therapeutics, Mirvie, Next Thought AI, Orange Street Ventures, Personalis, Protos Biologics, Qbio, RTHM, SensOmics. M. P. S. serves as a scientific advisor for Abbratech, Applied Cognition, Enovone, Jupiter Therapeutics, M3 Helium, Mitrix, Neuvivo, Onza, Sigil Biosciences, TranscribeGlass, WndrHLTH, Yuvan Research. M. P. S. is a co-founder of NiMo Therapeutics. M. P. S. is an investor and scientific advisor of R42 and Swaza. M. P. S. is an investor in Repair Biotechnologies. The other authors declare no competing interest.

## Supplemental data

Table S8

Figure SF1 **Preparing chromatin for analysis.** (*A*) bright field and GFP fluorescence response to 21 h doxycycline treatment before (left, scale bar 400 μm) and after (*right*, scale bar 200 μm) monoclonal isolation by FACS. Approximately 5% of polyclonal cells responded to doxycycline treatment before FACS as measured by GFP fluorescence, indicated by the FACS report (*center*).(*B*) Representative chromatin size distribution by bioanalyzer analysis after sonication for cross-linked chromatin for ChIP-qPCR. (*C*) Representative chromatin size distribution by bioanalyzer analysis after sonication for non-cross-linked chromatin for proteomic analysis.

Figure SF2 **Total ion chromatograms from each high pH fraction from 2D-LC.** TICs produced with RaMS package in R (107) from the.mzML file corresponding to each high pH fraction. ChIP, chromatin immunoprecipitation; ENCODE, Encyclopedia of DNA Elements; FACS, fluorescence activated cell sorting; TF, transcription factor; TPM, transcripts per million; TSS, transcription start site.
