# Supplementary material for: In Depth Characterization of the Promoter Proximal Proteome of Single Copy Locus FOXP2
Source: Mol Cell Proteomics. 2026 Apr 21;25(6):101570. doi: 10.1016/j.mcpro.2026.101570 (PMC13227211; doi:10.1016/j.mcpro.2026.101570)
Supplement: Figure SF1 — Preparing chromatin for analysis. (A) bright field and GFP fluorescence response to 21 h doxycycline treatment before (left, scale bar 400 μm) and after (right, scale bar 200 μm) monoclonal isolation by FACS. Approximately 5% of polyclonal cells responded to doxycycline treatment before FACS as measured by GFP fluorescence, indicated by the FACS report (center).(B) Representative chromatin size distribution by bioanalyzer analysis after sonication for cross-linked chromatin for ChIP-qPCR. (C) Representative chromatin size distribution by bioanalyzer analysis after sonication for non-cross-linked chromatin for proteomic analysis. [file mmc9.pdf]

SF1A

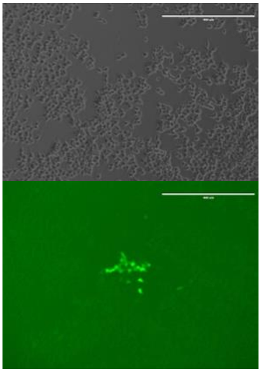

Tube: Caspex_DAPI_sort

Population	#Events	%Parent	%Total
All Events	50,000	####	100.0
scatter	42,052	84.1	84.1
singlets - ssc	40,676	96.7	81.4
singlets - fsc	39,747	97.7	79.5
live	36,551	92.0	73.1
GFPhi	502	1.4	1.0
GFPlo	2,854	7.8	5.7

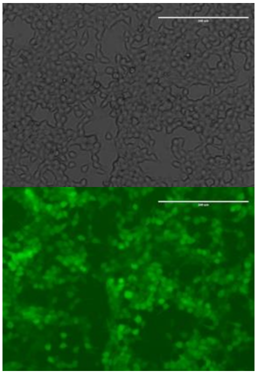

B

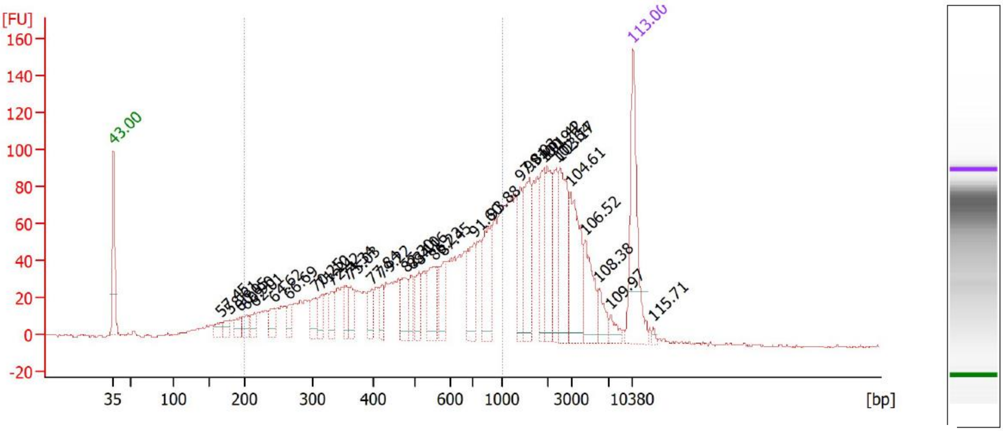

From [bp]	To [bp]	Corr. Area	% of Total	Average Size [bp]	Size distribution in CV [%]	Conc. [pg/µl]	Molarity [pmol/l]
200	1,000	1,343.3	54	536	39.5	945.30	3,419.6

C

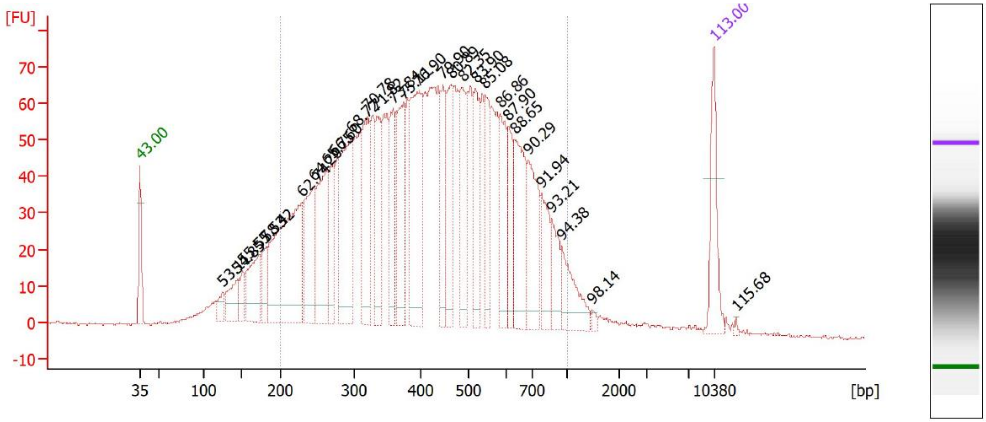

From [bp]	To [bp]	Corr. Area	% of Total	Average Size [bp]	Size distribution in CV [%]	Conc. [pg/µl]	Molarity [pmol/l]
200	1,000	2,378.8	88	447	36.8	4,284.09	17,513.2

Figure SF1. Preparing Chromatin for Analysis
(a) Bright field and GFP fluorescence response to 21 hr doxycycline treatment before (left, scale bar 400 µm) and after (right, scale bar 200 µm) monoclonal isolation by FACS. Approximately 5% of polyclonal cells responded to doxycycline treatment before FACS as measured by GFP fluorescence, indicated by the FACS report (center).
(b) Representative chromatin size distribution by Bioanalyzer analysis after sonication for cross-linked chromatin for ChIP-qPCR.
(c) Representative chromatin size distribution by Bioanalyzer analysis after sonication for non-cross-linked chromatin for proteomic analysis.
