# Supplementary material for: In Depth Characterization of the Promoter Proximal Proteome of Single Copy Locus FOXP2
Source: Mol Cell Proteomics. 2026 Apr 21;25(6):101570. doi: 10.1016/j.mcpro.2026.101570 (PMC13227211; doi:10.1016/j.mcpro.2026.101570)
Supplement: Figure SF2 — Total ion chromatograms from each high pH fraction from 2D-LC. TICs produced with RaMS package in R (107) from the.mzML file corresponding to each high pH fraction. ChIP, chromatin immunoprecipitation; ENCODE, Encyclopedia of DNA Elements; FACS, fluorescence activated cell sorting; TF, transcription factor; TPM, transcripts per million; TSS, transcription start site. [file mmc10.pdf]

SF2

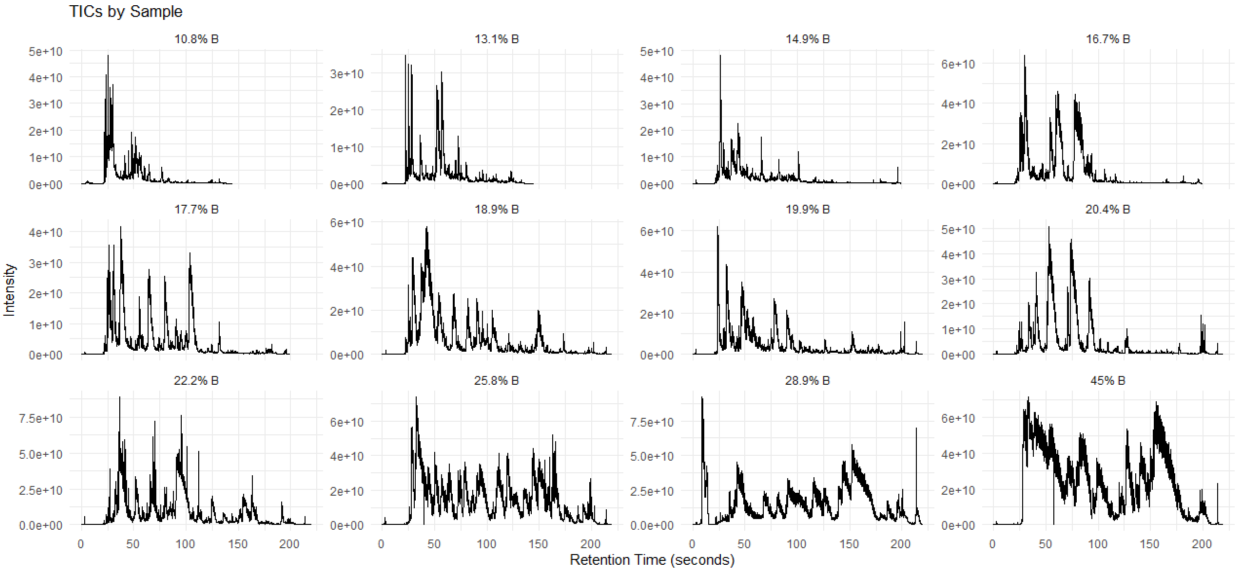

Figure SF2. Total Ion Chromatograms from Each High pH Fraction from 2D-LC

TICs produced with RaMS package in R [105] from the .mzML file corresponding to each high pH fraction.
